# Immunopathological investigation and genetic evolution of *Avian leukosis virus* Subgroup-J associated with myelocytomatosis in broiler flocks in Egypt

**DOI:** 10.1186/s12985-024-02329-7

**Published:** 2024-04-10

**Authors:** Ahmed Fotouh, Eman Abd El-Menamm Shosha, Ali Mahmood Zanaty, Marwa Mostafa Darwesh

**Affiliations:** 1 Pathology and Clinical Pathology Department, Faculty of Veterinary Medicine, New Valley University, Kharga, Egypt; 2 Virology Department, Faculty of Veterinary Medicine, New Valley University, Kharga, Egypt; 3 Gene Analysis Unit, Reference Laboratory for Quality Control on Poultry, Animal Health Institute, Agriculture Research Center (ARC), Giza, Egypt; 4 Department of Pathology, Faculty of Veterinary Medicine, Benha University, Moshtohor, Toukh, 13736 Qaluiobiya Egypt

**Keywords:** *Avian leucosis virus*-J, Myelocytomatosis, PCR, Sequencing, Broilers, Pathology, Immunohistochemistry

## Abstract

**Background:**

*Avian leukosis virus* Subgroup-J (ALV-J) is a rapidly oncogenic evolving retrovirus infecting a variety of avian species; causing severe economic losses to the local poultry industry.

**Methods:**

To investigate ALV-J, a total of 117 blood samples and 57 tissue specimens of different organs were collected for virological, and pathological identification, serological examinations, molecular characterization, and sequencing analysis. To the best of our knowledge, this is the first detailed report recorded in broiler flocks in Egypt. The present study targets the prevalence of a viral tumor disease circulating in broiler flocks in the El-Sharqia, El-Dakahliya, and Al-Qalyubiyya Egyptian governorates from 2021 to 2023 using different diagnostic techniques besides ALV-J *gp85* genetic diversity determination.

**Result:**

We first isolated ALV-J on chicken embryo rough cell culture; showing aggregation, rounding, and degeneration. Concerning egg inoculation, embryonic death, stunting, and curling were observed. Only 79 serum samples were positive for ALV-J (67.52%) based on the ELISA test. Histopathological investigation showed tumors consist of uniform masses, usually well-differentiated myelocytes, lymphoid cells, or both in the liver, spleen, and kidneys. Immunohistochemical examination showed that the myelocytomatosis-positive signals were in the spleen, liver, and kidney. The PCR assay of ALV-J *gp85* confirmed 545 base pairs with only 43 positive samples (75.4%). Two positive samples were sequenced and submitted to the Genbank with accession numbers (OR509852–OR509853). Phylogenetic analysis based on the *gp85* gene showed that the ALV-J Dakahlia-2 isolate is genetically related to ALV-EGY/YA 2021.3, ALV-EGY/YA 2021.4, ALV-EGY/YA 2021.14, and ALV-EGY/YA 2021.9 with amino acid identity percentage 96%, 97%; 96%, 96%; respectively. Furthermore, ALV-J Sharqia-1 isolate is highly genetically correlated to ALV-EGY/YA 2021.14, and ALV-EGY/YA 2021.9, ALV-J isolate QL1, ALV-J isolate QL4, ALV-J isolate QL3, ALV-EGY/YA 2021.4 with amino acid identity percentage 97%, 97%; 98%, 97%, 97%, 95%; respectively.

**Conclusions:**

This study confirmed that ALV-J infection had still been prevalent in broilers in Egypt, and the genetic characteristics of the isolates are diverse.

**Supplementary Information:**

The online version contains supplementary material available at 10.1186/s12985-024-02329-7.

## Background

*Avian leucosis* virus (ALV) is an RNA virus belonging to the genus *Alpharetrovirus*, family *Retroviridae*, and induces a lot of neoplastic diseases with other reproduction troubles in different poultry species worldwide [1, 2]. *Avian leucosis* virus type J (ALV-J), strain HPRS-103, was first isolated from commercial meat-type chickens in the United Kingdom in the late 1980s [1, 3]. Moreover, in China, ALV-J infection was first detected in 1999 then followed by devastation to layers, of native breeds of chickens and ducks, causing catastrophic economic losses [4]. To date, ALVs have been classified into 11 viral subgroups, based on their host range, as well as viral envelope interference and cross-neutralization patterns [5]. Subgroups A-D, K, and J are exogenous viruses; that mainly infect chickens and turkeys, whereas subgroup E belongs to endogenous viruses. Subgroups ALV-A and ALV-B are common pathogens inducing lymphoid leukosis tumors with high incidence, while subgroups ALV-C and ALV-D have rarely affected the chicken [6]. Recently, subgroup ALV-K induces fowl glioma [7].

Importantly, chickens infected with ALV-J usually exhibit tumor development, depressed immunity, growth retardation, reduced egg productivity, and a considerable increase in the morbidity and mortality rates that are most apparent in broiler breeder hens. ALV-J infection in breeder flocks is associated with the occurrence of myeloid leucosis (myelocytomatosis) [8]. Myelocytomatosis was first observed in broiler breeder birds between 25 and 55 weeks. Furthermore, myelocytomatosis tumors are now being reported to appear in the field as early as 17 weeks [5, 9]. Numerous further cases of myelocytomatosis among broiler breeding flocks have also been reported in several European countries [10]. America, Asia [4], Africa (Egypt) [11], and Australia [12].

In recent years, numerous strains of ALV Subgroup-J have been isolated from white meat-type chickens; causing a serious impact on the growth performance of the poultry industry [3]. Due to horizontal and vertical transmission, ALV-J has caused increasingly severe damage to the poultry industry worldwide as infected broilers exhibit various tumor phenotypes, such as hemangioma, myeloid leukosis, and myelocytoma with decreased weight gain [13, 14]. Moreover, in other parts of the world as China, the mixed structure of the chicken breeding industry through crossbreeding Chinese local breeds with other western breeds may potentially be of concern in increasing the high frequency of ALV-J infection, especially in the case of vertical transmission to their progeny [15]. In Egypt, ALV-J-induced myelocytomatosis was reported in broilers − 28 days old-depending on histopathological lesions and antibody detection as the positive samples were (26%) with a mortality rate was (3.5%) [11, 16]. Taken into account, myelocytomatosis diagnosis is based on gross pathological lesions and antibody titer in a 27-week-old broiler breeder. Additionally, in laying hens, ALV-J particles are presented abundantly in the ovarian stroma, bud from cells in direct contact with oogonia, and oocytes with the highest concentration in the albumen-secreting glands of the magnum. This early ovarian and oviduct exposure may cause an early and diffuse infection [17]. Moreover, monoclonal antibodies against ALV-J envelop glycoproteins have been produced with broad reactivity for most ALV-J isolates. These antibodies have been used to determine tissue tropism of ALV-J naturally infected chickens [18].

Currently, there is no vaccination strategy or specific treatment available for ALV-J. Thus, control measures depended on the elimination of positive cases, management approaches and biosecurity programs in the poultry farms to decrease ALV spreading and clinical symptoms [19]. Therefore, rapid and confirmatory diagnosis is necessary to eradicate ALV from breeding flocks [20]. The ELISA is a useful serological diagnosis for the detection of ALV-J antibodies as it is a sensitive, easy, safe, and rapid diagnostic tool [11]. ELISA diagnostic method of ALV-J is reported to have sensitivity (99.2%), and specificity (100%), and can be used clinically for screening purposes [21].

Recently, molecular-based diagnostic techniques including *insitu* hybridization, PCR, and sequencing analysis have been developed for ALV detection [22]. Particularly, the proviral DNA arrangement of the ALV genome involves three important structural proteins (gag, pol, and env) which have been translated into the specific group antigen and envelop glycoproteins. The *gp85* envelop glycoprotein, is closely associated with the viral entry, and host range, inducing host-neutralizing antibodies, tissue tropism, and virulence. Moreover, it is the major subgrouping determinant responsible for host infection and tumor formation [23–25]. In addition, the *gp85* gene is the most variable region of the envelope which evolves more rapidly in ALV-J compared to the other subgroups causing serious economic losses. Thus, it is crucial to monitor the *gp85* gene evolution continuously to update any new strains and mutations [19, 26, 27].

Taken together, the present study targets the molecular characterization, serological assay, and sequencing analysis of ALV-J isolates that are circulating in broiler flocks in El-Sharqia, El-Dakahliya, and Al-Qalyubiyya Egyptian governorates through PCR technique, ELISA, and molecular sequencing approaches. Our study also involved the myelocytomatosis diagnosis in naturally infected broiler chickens with a complete pathological and immunohistochemical picture of different infected organs.

## Materials and methods

### History of examined farms and sampling

A total number of 18 broiler farms of different breeds, aged from 5 to 7 weeks old, were investigated during the period from November 2021 to April 2023. Capacity of farms ranged from 5000 to 15,000 birds/farm. The broiler farms were located in the Lower Egypt region in 3 Governorates; El-Sharqia, El-Dakahliya, and Al-Qalyubiyya (Table 1). Collectively, a total of 117 blood samples were collected from suspected diseased birds for serological examination using the ELISA test. Besides, all positive samples for the ELISA test are recommended for PCR tests and histopathological examination, thus we selected a total of 57 tissue specimens of positive cases including liver, spleen, and kidney. The sampled flocks were recently diseased birds that had shown depression, anorexia, and growth retardation.

**Table 1:** The descriptive flock data of the investigated broilers for ALV-J detection from 2021 to 2023 in the El-Sharqia, El-Dakahliya, and Al-Qalyubiyya Egyptian governorates

Flock location	Broiler farms	Flock age	No. of birds	Collected samples				Date of collection
				Blood	Liver	Spleen	Kidney	
Sharqia	6	5 weeks	9000–12,000	38	8	5	4	2021
El-Dakahliya	3	7 weeks	5000–7000	29	5	3	2	2023
Al-Qalyubiyya	9	6 weeks	12,000–15,000	50	10	11	9	2022

### Virus isolation assays

The chicken embryo rough (CER) cell line was purchased from VACSERA (Vaccine and Serum Association), Dokki, Giza, Egypt for virus isolation and culturing**.** Samples were processed and then inoculated in CER cells with continuous monitoring for the evidence of virus growth. The inoculated cells were incubated at 37 °C with 5% CO_2_ for 5 days for each passage. An uninfected CER cell line is considered a negative control. Cell cultures are noticed daily for any cytopathic effects (CPE) according to the method described by [16, 28]. After three serial cell passages, the culture supernatants containing the virus were harvested to confirm by PCR test [25]. Concerning embryonated chicken eggs (ECE) inoculation, the collected tissue specimens (liver, spleen, and kidney) were processed for virus isolation. The processed samples were inoculated in 9-day-old specific-pathogen-free (SPF)-ECE, then incubated at 37 °C for 5–7 days, after that examined daily by candling with recording any mortality cases. The embryos were opened aseptically for gross lesion examination [29]. Consequently, the amniotic fluid was harvested and then tested using specific PCR analysis.

### Serological assay

The collected blood samples, from the wing vein, were centrifuged at 3000 rpm for 15 min for serum separation. Sera were stored at − 20 °C until used for detection of the anti-p27 antibody developed against ALV-J in the serum of all diseased chickens using an antigen capture ELISA (ELISA Test Kit, IDEXX Laboratories, Inc., USA). The results interpretation was performed according to [16, 29]. In addition, the difference of optical density (OD) between antigen-coated wells and serum sample, in which the sample to the positive ratio (S/P) ratio was expressed as follows: S/P ratio = (OD of sample − OD of negative control)/(OD of positive control − OD of negative control). Samples with an S/P ratio of 0.6 or greater were considered positive.

### Proviral DNA extraction and PCR analysis

Tissue samples (liver, spleen, and kidney) were kept at − 70 °C until used for DNA detection of ALV-J by PCR amplification using Phusion^®^ High-Fidelity DNA Polymerase (Thermo, MA, USA) regarding the manufacturer’s methods. The DNA extraction was carried out according to [30]. The oligonucleotide primers used in PCR amplification of ALV-J included; the forward primer H5 was annealed from the 3′ region of the pol gene which was conserved mainly across ALV subgroups. The reverse primer H7 was annealed specifically from a well-conserved region of the *gp85* of ALV-J. This pair of primers for ALV-J gives a PCR amplification product size in 545 base pairs (bp) [31]. The primers H5 and H7 originated from the HPRS-103 ALV-J prototype strain (Genbank accession No. Z46390) (Table 2).

**Table 2:** Sequence of oligonucleotide primers, targets, and expected PCR product sizes

Primer for ALV-J	Sequence (5′–3′)	Position	Product size with H5 (bp)	Reference
H5-F	5′-GGATGAGGTGACTAAGAAAG-3′	5258–5277	545	Smith et al. [31]
H7-R	5′-CGAACCAAAGGTAACACACG-3′	5783–5802		

### DNA sequencing and phylogenetic analysis

The two positive amplicons were completely purified using the QIAquick Gel Purification Kit (Qiagen, Hilden, Germany), then sequenced using the BigDye Terminator v3.1 Cycle sequencing Kit (Applied Biosystems, California, USA) via specific primers for *gp85* of ALV-J. The oligonucleotide sequence was established using ABI 3500 Genetic Analyzer (Life Technologies, California, USA). The nucleotide and amino acid sequences were aligned with other related strains in Genbank using the Clustal W program. A phylogenetic tree was designed using the MEGA-X program [32] and BioEdit software packages, with levels assessed using 1000 bootstrap replicates [33]. The sequences of the ALV-J strains in Genbank isolated from broilers, layers, and breeders were comprised in the multiple-sequence alignment and then summarized in (Table 3).

**Table 3:** ALV-J referential strains used in this study

Number (No.)	Strains	Origin	Accession no	Subgroup
1	Dakahlia-2-ALV-J	EGY(this study)	OR509852	J
2	Sharqia-1-ALV-J	EGY (this study)	OR509853	J
3	HPRS-103	UK	Z46390	J
4	ADOL-7501	USA	AY027920	J
5	ADOL-R5-4	USA	AF076887	J
6	UD5	USA	AF307952	J
7	UD4	USA	AF307951	J
8	UD3	USA	AF307950	J
9	UD2	USA	AF307949	J
10	10022-2	USA	GU222396	J
11	10022-16	USA	GU222400	J
12	10022-20	USA	GU222401	J
13	6803	USA	AF247388	J
14	AF88	USA	AF247390	J
15	1696	USA	AF247384	J
16	RAV-1	USA	MF926337	A
17	Ev-1	USA	AY013303 E	E
18	CLB908U	RUS	JQ935966	J
19	MRL905	RUS	JF951728	J
20	SVR807	RUS	HM776937	J
21	MRL905	RUS	JF951728	J
22	NG-VX29	NGA	MH669345	J
23	NG VX32	NGA	MH669346	J
24	EO49–1	NGA	MF926334	J
25	EO59	NGA	MF926336	J
26	QL1 eg	EGY	MN496121	J
27	QL2 eg	EGY	MN496122	J
28	QL3 eg	EGY	MN496123	J
29	QL4 eg	EGY	MN496124	J
30	QL5 eg	EGY	MN496125	J
31	QL6 eg	EGY	MN496126	J
32	TgM/00	EGY	DQ316907	J
33	EgM/00	EGY	DQ316906	J
34	YZ9902	CHN	HM235670	J
35	CAUHN01	CHN	HM640944	J
36	WLY13	CHN	KJ631311	J
37	JS-nt	CHN	HM235667	J
38	WGZ13	CHN	KJ631313	J
39	WL12	CHN	KJ631318	J
40	WJ612	CHN	KJ631317	J
41	WGD13	CHN	KJ631312	J
42	WC512	CHN	KJ631316	J
43	WA1112	CHN	KJ631315	J
44	NX0101	CHN	AY897227	J
45	HuB09WH02	CHN	HQ634804	J
46	JS09GY3	CHN	GU982308	J
47	HN1001–1	CHN	HQ260974	J
48	HN1001–2	CHN	HQ260975	J
49	YZ9901	CHN	NM2002–1	J
50	SD0101 2	CHN	AY897225	J
51	HLJ10SH03	CHN	HQ634813	J
52	HLJ09SH01	CHN	HQ634806	J
53	JL09H01	CHN	HQ148554	J
54	GD1109	CHN	JX254901	J
55	TW99	CHN	AF497905	J
56	GDQJ-1	CHN	KU254611	J
57	FJ201307	CHN	KM655821	J
58	SH18JY01	CHN	MN735306	J
59	SH18JY02	CHN	MN735307	J
60	SCSM00	CHN	KF796652	Not identified
61	HB18XH01	CHN	MN735298	Not identified
62	HB201101	CHN	MW476817	J
63	HB201103-1	CHN	MW476821	J
64	NM2002-1	CHN	HM235669	J
65	GX20YL12	CHN	MT512432	J
66	SDAU09E3	CHN	JF826241	B
67	SDAU09C3	CHN	HM452340	A
68	SDAU09E1	CHN	HM452341.1	A
69	GD14LZ	CHN	KU605774	K
70	SD0001	CHN	AY897223	J
71	NHH	CHN	HM235668	J
72	LN08SY10	CHN	HQ634802	J
73	GDFX0601	CHN	KP686142	K
74	JS11C1	CHN	KF746200	K
75	WB11098e	CHN	JX570792	E
76	WB11008e	CHN	JX570786	E
77	WB12062e	CHN	KJ009323	E
78	WB11110e	CHN	JX570797	E
79	Km-5845	JPN	AB670314	K
80	PK19FA01	PK	MN956379	J
81	PK19SA01	PK	MN956380	J
82	HPRS103 YSL/02	EGY	DQ316908	J
83	Egy/YA-2021.3	EGY	MZ614719	J
84	Egy/YA-2021.9	EGY	MZ614720	J
85	Egy/YA-2021.10	EGY	MZ614721	J
86	Egy/YA-2021.14	EGY	MZ614722	J
87	Egy/YA-2021.4	EGY	MZ614723	J

Our isolates from this study are 1–2. Isolates 3–4 are British and American ALV-J reference strains. Isolates 5–17 are American ALV-J reference strains. Isolates 18–21 are Russian ALV-J reference strains. Isolates 22–25 are Nigerian ALV-J reference strains. Isolates 26–33 are Egyptian ALV-J reference strains. Isolates 34–79 are Chinese ALV-J reference strains. Isolates 80 is a Japanese ALV-J reference strain. Isolates 81–82 are Pakistan ALV-J reference strains. Isolates 83–88 are Egyptian ALV-J reference strains. Origin of ALVs: USA = United States,UK = United Kingdom, PK = Pakistan, CHN = China, RUS = Russia, JPN = Japan, EGY = Egypt, and NGA = Nigeria

### Analysis of the recombination events

Recombination events of each ALV-J strain isolated in our study were investigated according to [34] (RDP-5 Program), the used algorisms including BootScan, MaxChi, GENECONV, SiScan, Chimaera, LARD, RDP5, Phyl-Pro, and 3Seq, were utilized for comparison [35]. Recombination events were maintained by four independent approaches or more; which were only considered as accurate positive events.

### Histopathological examination

Tissue specimens (lung, liver, spleen, heart, kidney, proventriculus, gizzard, and bone) were collected for histopathological examination. These tissue specimens were fixed with (10% neutral buffered formalin), washed, dehydrated, and embedded in paraffin. Paraffin blocks were sectioned at 5 µm thickness and stained with hematoxylin and eosin (H&E), and also Giemsa stain was used when needed [36].

### Immunohistochemical examination

To detect the presence of ALV-J antigen, tissues were fixed with 10% buffered neutral formalin, paraffin-embedded, sectioned with 4 microns’ thickness, and mounted on poly-l-lysine-coated slides. The tissue sections were stained with a routine streptavidin–biotin/horseradish peroxidase (HRP)-conjugated immunohistochemical technique. The sections were prepared and stained to be examined microscopically with light microscopy according to the method described by [37].

## Results

### Clinical findings and mortality incidence

Clinical signs in 18 broiler farms of different breeds, aged from 5 to 7 weeks old, in the Lower Egypt region were mostly non-specific. The sampled broiler flocks had shown depression, anorexia, growth retardation, weakness, and dehydration. The mortality rate was recorded at about 7% while the morbidity was about 20%.

### Virus isolation on tissue culture and SPF-ECE

**Fig. 1:**
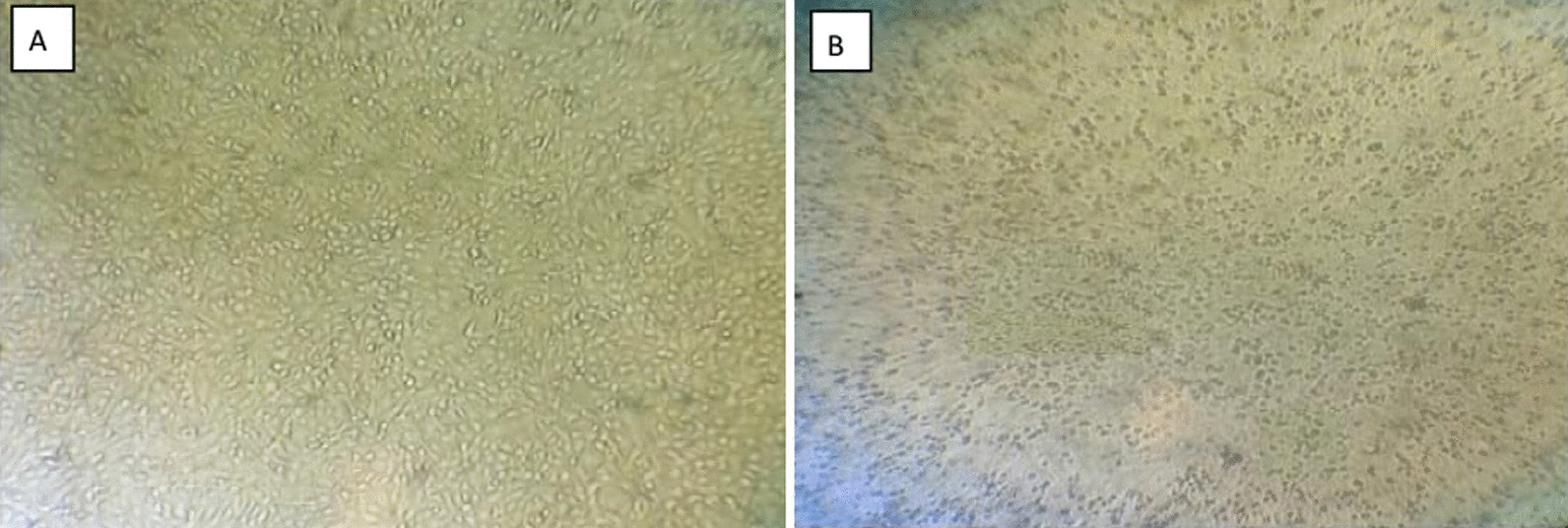
ALV-J isolation on CER cells. **A** The normal spindle uninoculated CER cells. **B** CER cells showaggregation, degeneration, and detachment of cells (Magnificent power is 10×)

**Fig. 2:**
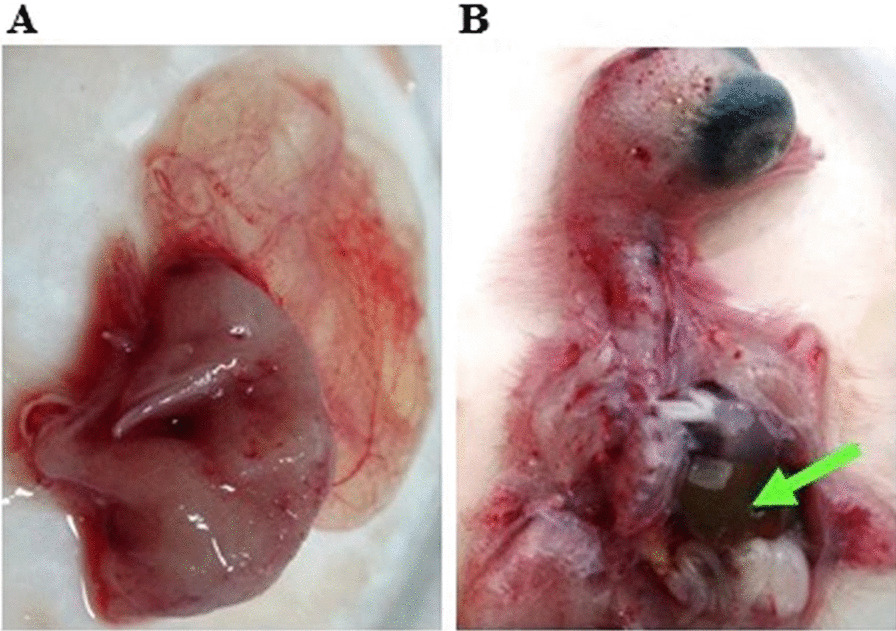
Macroscopic lesions from internal organs of an embryo with ALV-J associated with myelocytomatosis; **A** Chick an embryo showing an enlarged liver. **B** Chick embryo showing stunting and curling

The characteristic CPE does not appear up to the second passage level. The prominent CPE of inoculated ALV-J appears at the 3rd passage. Also, ALV-J CPE was observed after 72 h (h) in inoculated CER cell culture. CER cell culture showed aggregation which progressed rapidly; also rounding and degeneration (Fig. 1B). On the 5th day post-inoculation (d.p.i), there were enormous detachments of cells. Whereas, the uninfected control cells showed no changes (Fig. 1A). On the other hand, ALV-J was inoculated successfully in the SPF-ECE. After 7 days, mortality and gross pathological lesions of the inoculated embryos were observed; including embryonic death after 48 h p.i. and also the survived embryo on 7th d.p.i showed stunting, curling, dwarfing (Fig. 2B), anomalies, hemorrhages on the body surface, enlarged liver (Fig. 2A), and congestion of the chorioallantoic membrane (CAM).

### Antigen capture ELISA

The ELISA results are calculated based on the S/P ratio. Serum samples giving an S/P ratio of 0.6 or greater were considered positive. Collectively, serum samples of broiler farms at different ages revealed that among 117 serum samples, only 79 samples were positive for ALV-J (67.52%), with a mean of 0.95 for the S/P ratio of sera collected from diseased flocks. Meanwhile, 38 samples were negative for ALV-J (32.47%) with a mean of 0.38 for the S/P ratio of sera collected from healthy flocks (Additional file 1).

### Molecular identification

**Fig. 3:**
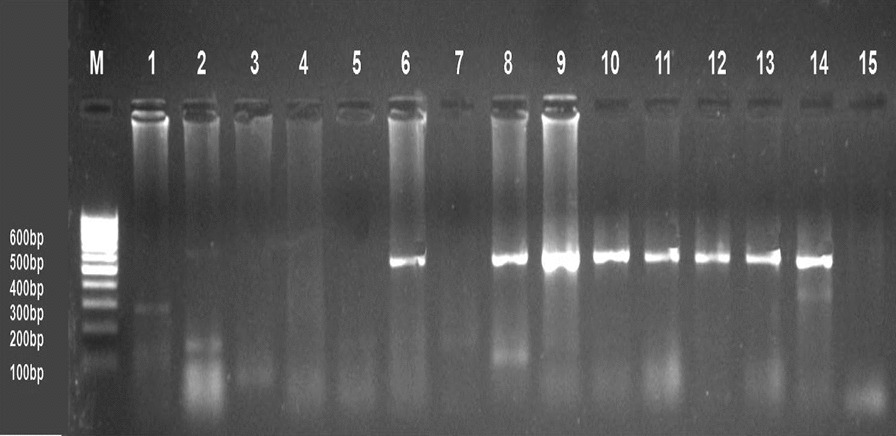
Ethidium bromide-stained 2% agarose gel of PCR products showed ALV-J. (−ve) samples; lanes 1–5 and 7, (+ve) samples; lanes 6, 8–13 of 545 bp PCR products, (+ve) control; Lane 14 and (−ve) control; Lane 15. M: represents a 100-bp ladder as a size standard

**Fig. 4:**
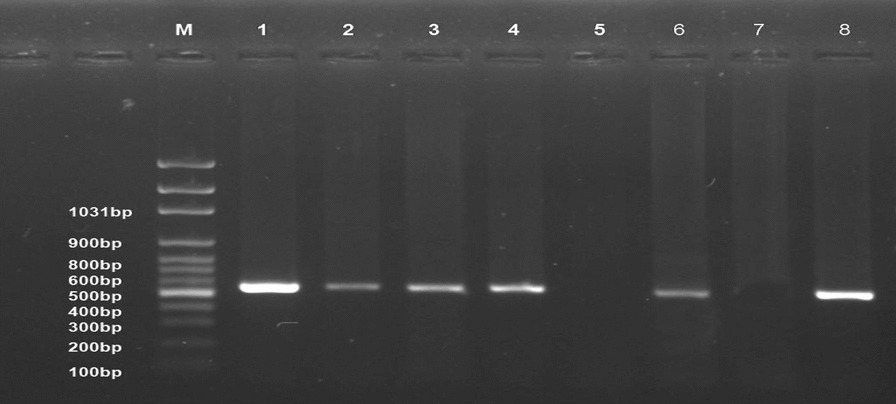
Ethidium bromide-stained 1% agarose gel of PCR products showed that ALV-J. Samples (1–3) were tissue culture and samples (4, 6) were Amniotic fluid. (+ve) samples; lanes (1–4) and 6 of 545 bp PCR products, (−ve) sample; lane 5, (−ve) control; Lane 7 and (+ve) control; Lane 8. M: represents a 100 bp ladder as a size standard

Collectively, 57 tissue samples (liver, spleen, and kidney) are examined from 14 farms with positive ELISA for ALV-J. PCR results showed that only 43 samples were positive (75.4%) (Fig. 3). These PCR results for different organs are summarized in (Table 4). The culture supernatants and amniotic fluid samples were tested by PCR as shown in (Fig. 4).

**Table 4:** Detection of ALV-J proviral DNA in different organs of broiler flocks using PCR

Organ	Samples no	Positive samples	
		No	%
Liver	23	21	91.3%
Spleen	19	17	89.4%
Kidney	15	5	33.3
Total	57	43	75.4

### Sequencing analysis of the ALV-J gp85 gene

**Fig. 5:**
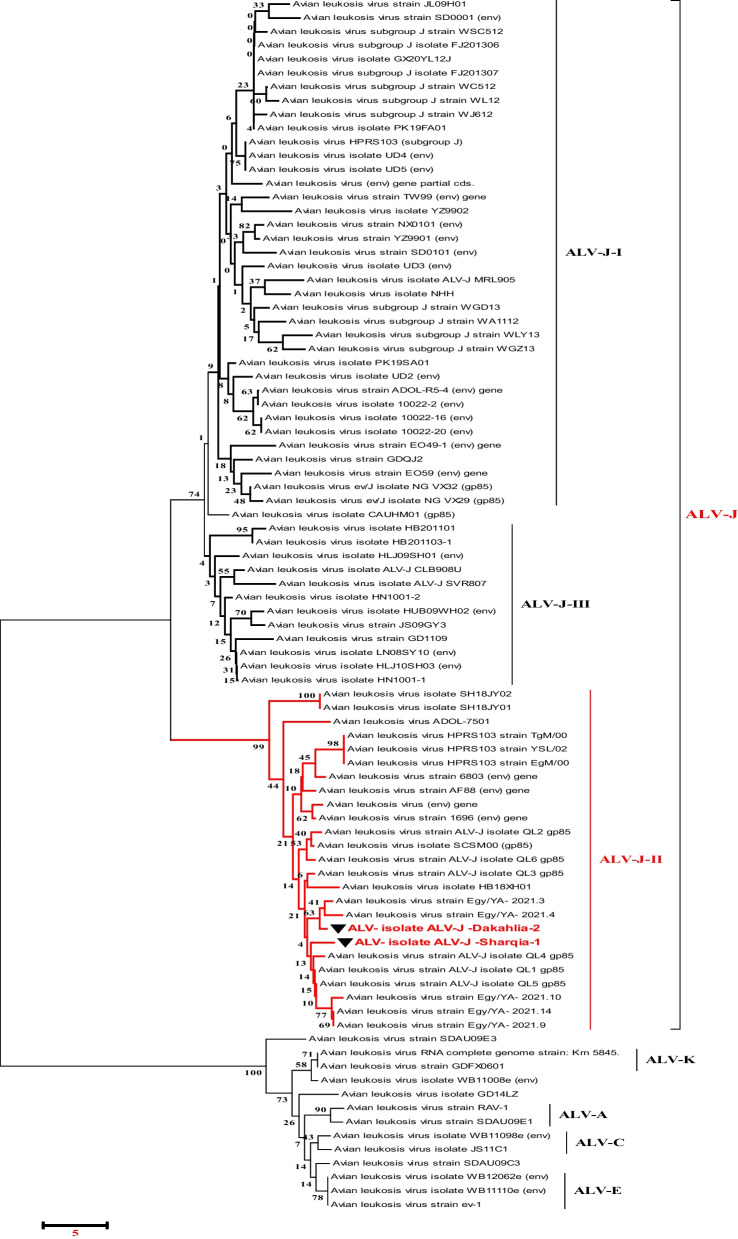
Collective phylogenetic tree based on gp85 gene sequences alignment of ALV-J with other reference sequences. The phylogenetic analysis of the ALV-J gp85 gene revealed that our ALV-J two isolates located in subgroups II (ALV-J Dakahlia-2 and ALV-J Sharquia-1) with other Egyptian strains cluster in the same group (Subgroups II). The ALV-J two isolates in our study are indicated by a triangle. The tree was constructed by the neighbor-joining method with 1000 bootstrap replicates, using MEGA 7.0

**Fig. 6:**
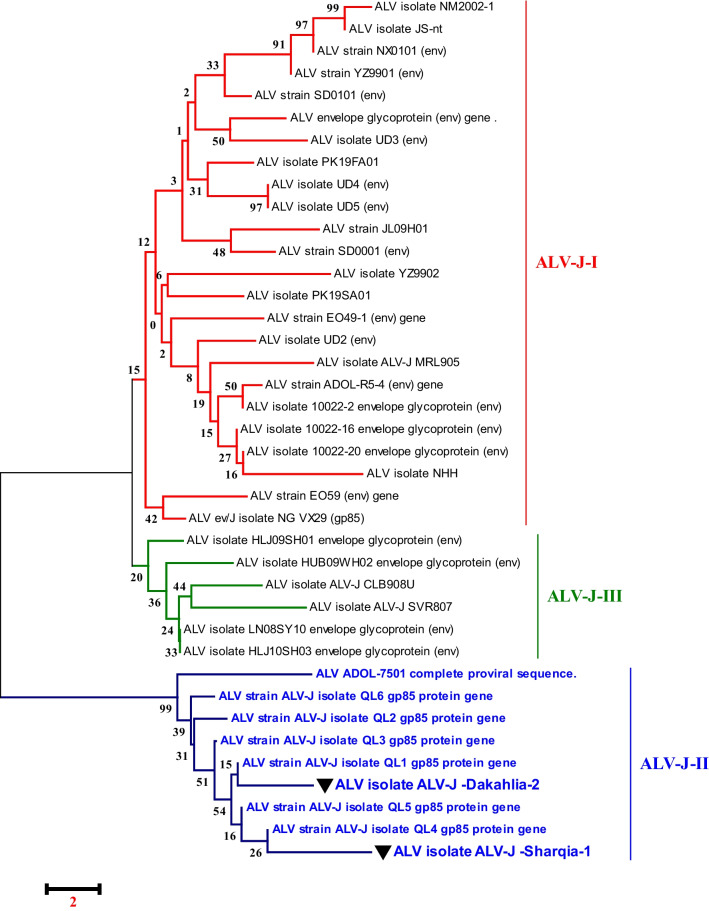
Detailed phylogenetic tree based on gp85 gene sequences alignment of ALV-J. This phylogenetic analysis of the ALV-J gp85 gene confirms that our ALV-J two isolates located in subgroup II are comparable to other strains deposited in Genbank

**Fig. 7:**
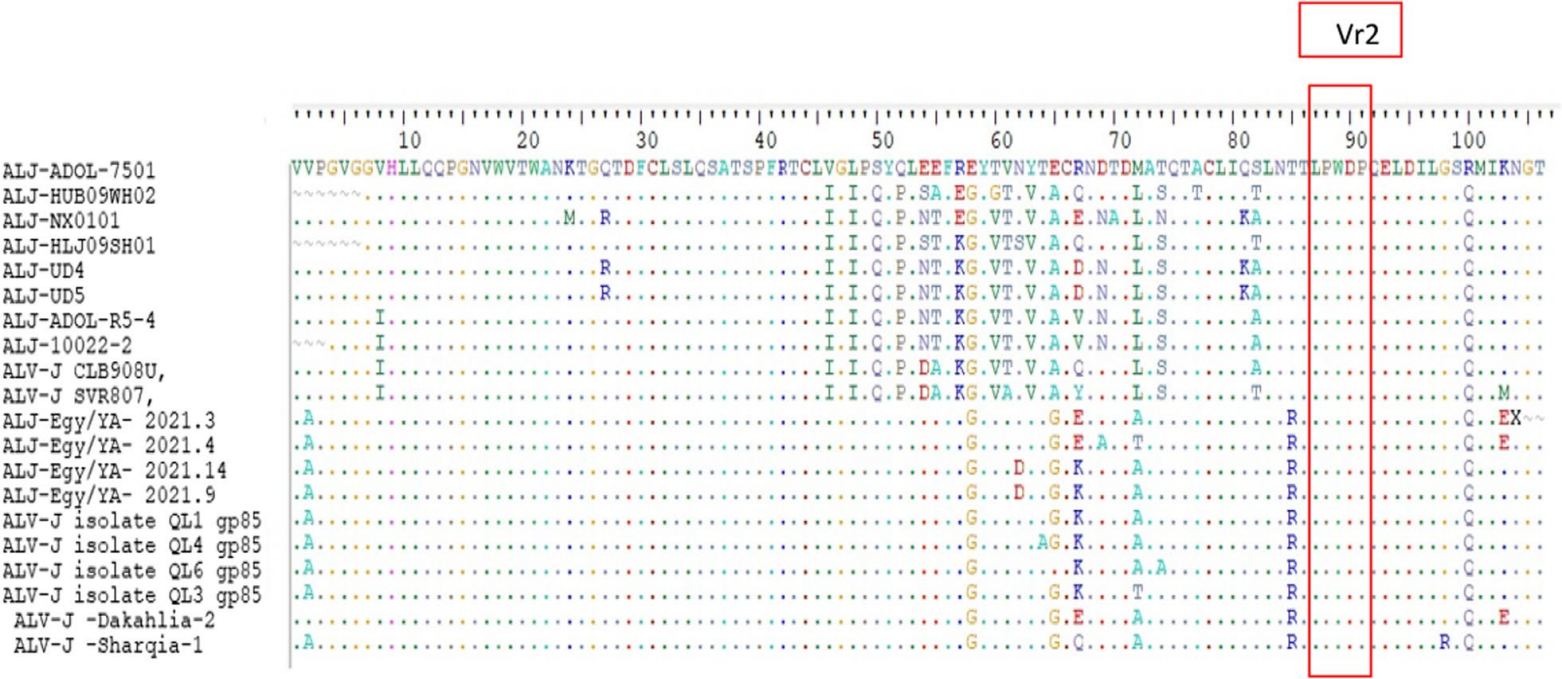
The gp85 sequence comparisons for the two ALV-J Egyptian isolates and reference strains. The top line represents the amino acid numbers in the gp85 sequence of HPRS-103. The letters indicate amino acid substitutions. The dots (.) indicate identical amino acids, dashes (−) indicate gaps produced in the alignment, and putative variable regions (Vr2) are indicated in red boxes and marked

Phylogenetic analysis was achieved using sequences from 88 ALV strains deposited in Genbank of various subgroups (Table 3). The phylogenetic trees construction based on the *gp85* gene sequences analysis (Figs. 5, 6), showed that Egyptian isolate (ALV-J Dakahlia-2, identified as subgroup II) (Fig. 6) have the highest genetically related to ALV-EGY/YA 2021.3, ALV-EGY/YA 2021.4, ALV-EGY/YA 2021.14, and ALV-EGY/YA 2021.9 (Egyptian isolates) with nucleotide identity percentage 100%, 97%, 96%, 96%; respectively, and on the amino acid level were with 96%, 97%; 96%, 96%; respectively (Table 5). Moreover, ALV-J Sharqia-1 isolate is highly genetically correlated to ALV-EGY/YA 2021.14, ALV-EGY/YA 2021.9, ALV-J isolate QL1, ALV-J isolate QL4, ALV-J isolate QL3, and ALV-EGY/YA 2021.4 (Egyptian isolates) with nucleotide identity percentage 98%, 98%, 98%, 98%, 98%, 97%; respectively, and on the amino acid level were with 97%, 97%; 98%, 97%, 97%, 95%; respectively. Also, ALV-J Dakahlia-2 and ALV-J Sharqia-1 isolates shared 91%, and 93% identity with ALJ-ADOL-7501 (American reference strain). Whereas, ALV-J isolates were distinctly apparent from ALJ-HUB09WH02 and ALJ-HLJ09SH01(Chinese isolates) sharing 71% and 73% similarity. In addition, ALV-J Dakahlia-2 and ALV-J Sharqia-1 isolates have low homology with ALJ-10022-2 in the USA, and ALV-J SVR807 in Russia with a percentage of 73%-75%, and 75%-77%; respectively (Table 5). Our Egyptian ALV-J isolates (ALV-J Dakahlia-2 and ALV-J Sharqia-1) were submitted to Genbank with accession numbers (OR509852–OR509853). Amino acid sequencing analysis of the *gp85* gene of our two isolates revealed 96% similarity to each other, also 96% similarity based on nucleotide identity level. In particular, no evidence of variations or amino acid mutations of our isolates in the *gp85* gene were detected in the putative variable regions, vr2 (Fig. 7). Regarding prototype strain HPRS103 (UK) and ADOL-7501 (USA) reference strains comparison, the sequencing analysis in our study clustered our two ALV-J isolates into subgroups II with other strains, as shown in (Fig. 6).

**Table 5:** Nucleotide identities and divergence of sequenced virus isolates comparable to other selected strains

Sample ID	ALJ-ADOL-7501	ALJ-HUB09WH02	ALJ-NX0101	ALJ-HLJ09SH01	ALJ-UD4	ALJ-UD5	ALJ-ADOL-R5-4	ALJ-10022–2	ALV-J CLB908U	ALV-J SVR807	ALJ-Egy/YA- 2021.3	ALJ-Egy/YA- 2021.4	ALJ-Egy/YA- 2021.14	ALJ-Egy/YA- 2021.9	ALV-J isolate QL1	ALV-J isolate QL4	ALV-J isolate QL6	ALV-J isolate QL3	ALV-J -Dakahlia-2	ALV-J -Sharqia-1	
Nucleotide identity %																					
	1 (%)	2 (%)	3 (%)	4 (%)	5 (%)	6 (%)	7 (%)	8 (%)	9 (%)	10 (%)	11 (%)	12 (%)	13 (%)	14 (%)	15 (%)	16 (%)	17 (%)	18 (%)	**19 (%)**	**20 (%)**	
1. ALJ-ADOL-7501		74	78	75	80	80	80	77	80	80	90	93	93	94	95	94	94	95	**91**	**93**	1
2. ALJ-HUB09WH02	77		89	98	89	89	89	92	90	89	71	72	73	73	74	72	73	74	**71**	**73**	2
3. ALJ-NX0101	79	83		89	97	97	95	92	94	93	74	76	76	76	77	76	76	77	**74**	**76**	3
4. ALJ-HLJ09SH01	77	95	84		91	91	90	93	90	90	71	73	73	73	75	73	74	74	**71**	**73**	4
5. ALJ-UD4	81	85	95	88		100	97	94	95	94	75	77	77	77	79	77	78	78	**76**	**77**	5
6. ALJ-UD5	81	85	95	88	100		97	94	95	94	75	77	77	77	79	77	78	78	**76**	**77**	6
7. ALJ-ADOL-R5-4	82	86	92	89	96	96		97	96	95	76	77	77	77	78	77	77	78	**76**	**77**	7
8. ALJ-10022–2	79	88	90	91	93	93	97		93	93	73	75	75	75	76	75	75	76	**73**	**75**	8
9. ALV-J CLB908U	83	89	90	90	93	93	96	93		97	75	77	78	78	79	78	78	79	**76**	**78**	9
10. ALV-J SVR807	82	87	87	88	91	91	93	91	96		75	77	77	77	79	77	78	78	**75**	**77**	10
11. ALJ-Egy/YA. 2021.3	90	75	76	75	77	77	78	76	79	79		97	97	96	95	95	94	95	**100**	**96**	11
12. ALJ-Egy/YA. 2021.4	92	76	79	76	80	80	81	79	81	81	95		98	98	97	97	96	97	**97**	**97**	12
13. ALJ-Egy/YA. 2021.14	92	77	78	78	80	80	81	79	82	81	94	95		99	98	98	97	98	**96**	**98**	13
14. ALJ-Egy/YA. 2021.9	92	77	78	78	80	80	81	79	82	81	94	95	100		98	98	97	98	**96**	**98**	14
15. ALV-J isolate QL1	93	78	79	78	81	81	82	80	83	82	95	96	99	99		98	98	99	**95**	**98**	15
16. ALV-J isolate QL4	92	77	78	77	80	80	81	79	82	81	94	95	98	98	99		98	98	**95**	**98**	16
17. ALV-J isolate QL6	93	78	79	78	81	81	82	80	83	82	93	94	97	97	98	97		98	**94**	**97**	17
18. ALV-J isolate QL3	93	78	79	78	81	81	82	80	83	82	94	97	98	98	99	98	97		**95**	**98**	18
19. ALV-J -Dakahlia-2	**93**	**77**	**80**	**77**	**81**	**81**	**82**	**79**	**83**	**83**	**96**	**97**	**96**	**96**	**97**	**96**	**95**	**96**		**96**	19
20. ALV-J -Sharqia-1	**92**	**78**	**78**	**78**	**80**	**80**	**81**	**79**	**83**	**81**	**94**	**95**	**97**	**97**	**98**	**97**	**96**	**97**	**96**		20
Amino acids identity %																					

Bold values refer to our ALV-J Egyptian samples

Nucleotide identities and divergence of sequenced virus isolates comparable to other selected strains from China, Russia, Egypt, the United Kingdom, and the USA. The figure reveals a comparative alignment of the gp85 gene in which, the gp85 nucleotide identity percentage of two of our Egyptian isolates ranges from 71 to 100% comparable to other different reference strains. Besides, the amino acids identity percentage of two of our Egyptian isolates ranges from 77 to 98% comparable to other different reference strains

### Analysis of the recombination events

According to the RDP5 Program, the recombination events in the *gp85* gene sequence of the two ALV-J Egyptian isolates were analyzed. There is no evidence of recombination detected in two ALV-J isolates (ALV-J Sharquia-1 and ALV-J Dakahlia-2).

### Pathological findings

#### Macroscopical findings

**Fig. 8:**
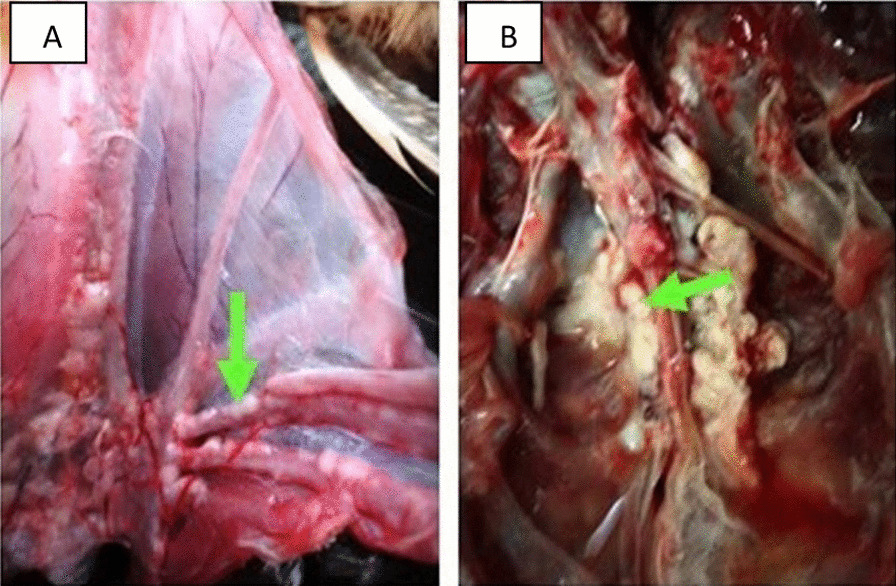
Macroscopic lesions from internal organs of broiler chickens naturally infected with myelocytomatosis; **A** (left): Costo-chondral junction of 7 weeks old broiler chicken showing whitish myelocytomas (arrow), and **B** (right): Pelvic bone of 7 weeks old broiler chicken showing whitish myelocytomas on the inner surface (arrow)

Necropsy mostly was not clear except in some cases showed off-white masses on the inner surface of both the sternum and pelvis (Fig. 8a, b). The tumors occur as soft and friable.

#### Microscopic findings

**Fig. 9:**
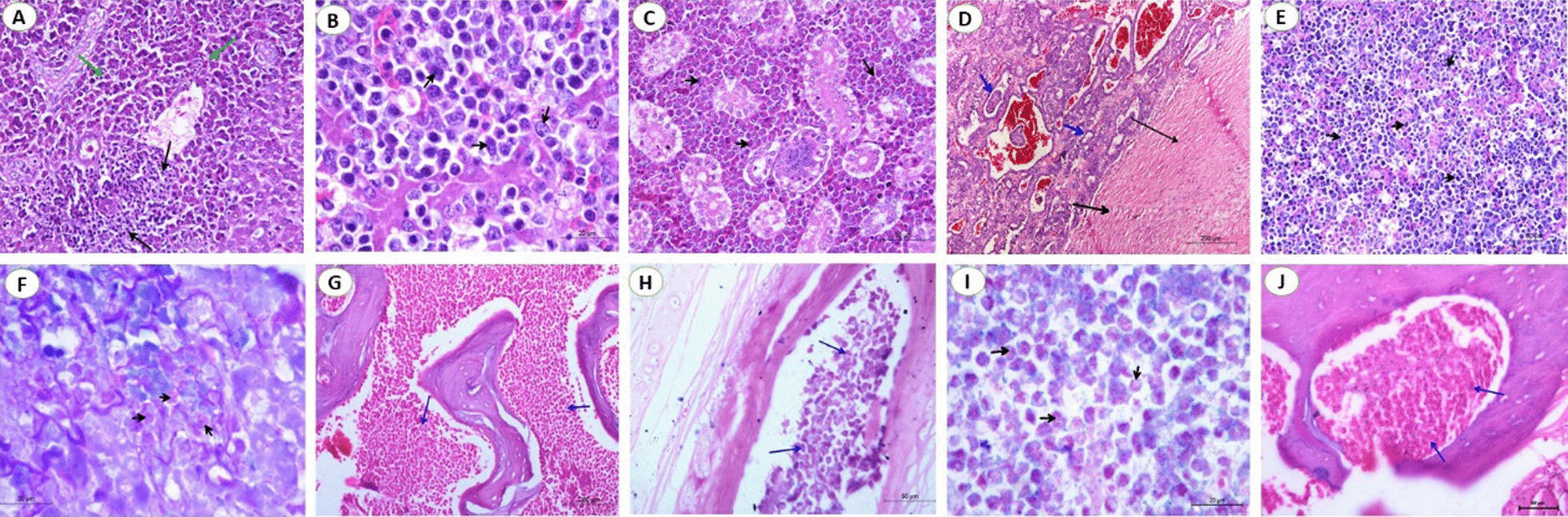
Photomicrograph sections from myelocytomatosis in liver, kidney, spleen, bone, and bone marrow of naturally infected broiler chickens. **A** Liver showing myeloid and lymphoid cell infiltrations (H&E, scale bar: 50 µm), **B** Liver showing lymphoblastic cells infiltration which are large, homogenous in size with poorly defined cytoplasmic membrane and basophilic cytoplasm (H&E, scale bar: 20 µm), **C** Kidney showing neoplastic aggregations of granular myeloid cells around renal tubules (arrow) and glomerulus (H&E, scale bar: 50 µm), **D** Nephrobastoma showing very dense fibrous capsule, the stromal tissue showed ill distinct glomeruli. Tumor tissue showed undifferentiated cystic renal tubules. The tubules are lined by multilayer epithelial cells (H&E, scale bar: 200 µm), **E** Spleen showing diffuse proliferation of large lymphoid cells in the red pulp (H&E, Scale bar: 50 µm), and **F** Spleen showing infiltration of myeloid cells in white pulp (arrow) (Giemsa stain, Scale bar: 20 µm). **G** Skull bone showing proliferation of granulated myelocytes in the bone marrow (arrow) (H&E, Scale bar: 200 µm), **H** Sternum bone showing proliferation of granulated myelocytes in the bone marrow (arrow) (Giemsa stain, Scale bar: 50 µm), **I** Myelocytoma; the cytoplasm of tumor cells were slightly basophilic and contained eosinophilic pinkish granules (Giemsa stain, Scale bar: 20 µm), and **J** Sternum bone showing proliferation of granulated myelocytes in the bone marrow (H&E, Scale bar: 50 µm)

*Liver* Tumors consist of masses of uniform, usually well-differentiated, myelocytes. Their nuclei appear as large, distinct, vesicular, and eccentrically located. The cytoplasm is saturated with the acidophilic spherical granules. The hepatic lobules showed multifocal aggregations of mature granulated myeloid cells with mitotic figures. The hepatocytes appeared as solitary islets inside the massive infiltration of myeloblastic cells. In some cases, myeloid and lymphoid tumors were detected in the same hepatic tissue (Fig. 9A). The lymphoblastic cells are large mononuclear cells homogenous in size with poorly defined cytoplasmic membrane and basophilic cytoplasm. The nuclei appeared vesicular in which margination and clumping of chromatin with the appearance of one or more obvious acidophilic nucleoli. These neoplastic cells showed mitotic figures (Fig. 9B).

*Kidneys* The renal tubules were separated from each other by massive infiltration of lymphoblastic cells. Renal tubules adhering to neoplastic cell masses suffer from degenerative changes and pressure atrophy. Diffuse granulated myeloid cells were seen to infiltrate the renal parenchyma causing pressure atrophy and loss of renal tubules (Fig. 9C). Nephroblastoma is capsulated by a dense fibrous capsule, the stromal tissue shows ill distinct glomeruli. Tumor tissue showed undifferentiated cystic renal tubules. The tubules are lined by multilayer epithelial cells. Most of the tubules contain hemorrhagic blood. The epithelial cells penetrating the connective tissue storms forming newly formed tubules (Fig. 9D).

*Spleen* Proliferation of lymphocytes that show pleomorphism, atypism, and mitotic activity. Diffuse proliferation of large lymphoid cells allover splenic splenic tissue especially red pulp was seen in some cases (Fig. 9E). Few myeloid cell infiltrations together with lymphoid cells were recorded (Fig. 9F). Proliferation of reticular cells which appear stellate, elliptical, fusiform, or spindle shape, and the nucleus takes the same shape of the cell (Additional file 1).

*Bone and myelocytoma* Proliferation of granulated myelocytes in the bone marrow of various bones and in the periosteum of the ribs, pelvis, and skull (Fig. 9G, H) and sternum (Fig. 9J). Proliferation begins in the bone marrow of epiphysis. The myelocytes invaded from the bone marrow to periosteal areas through Haversian and Volkmann’s canals. Additionally, there is a wall thickening and lumen narrowing of the sternal bone together with dentation in the periosteum which is considered osteopetrosis. The sections taken from the tumorous growths over the ribs, sternum, and pelvic bones revealed a marked proliferation of solid masses of uniform mature myelocytes (Fig. 9I).

### Immunohistochemical findings

**Fig. 10:**
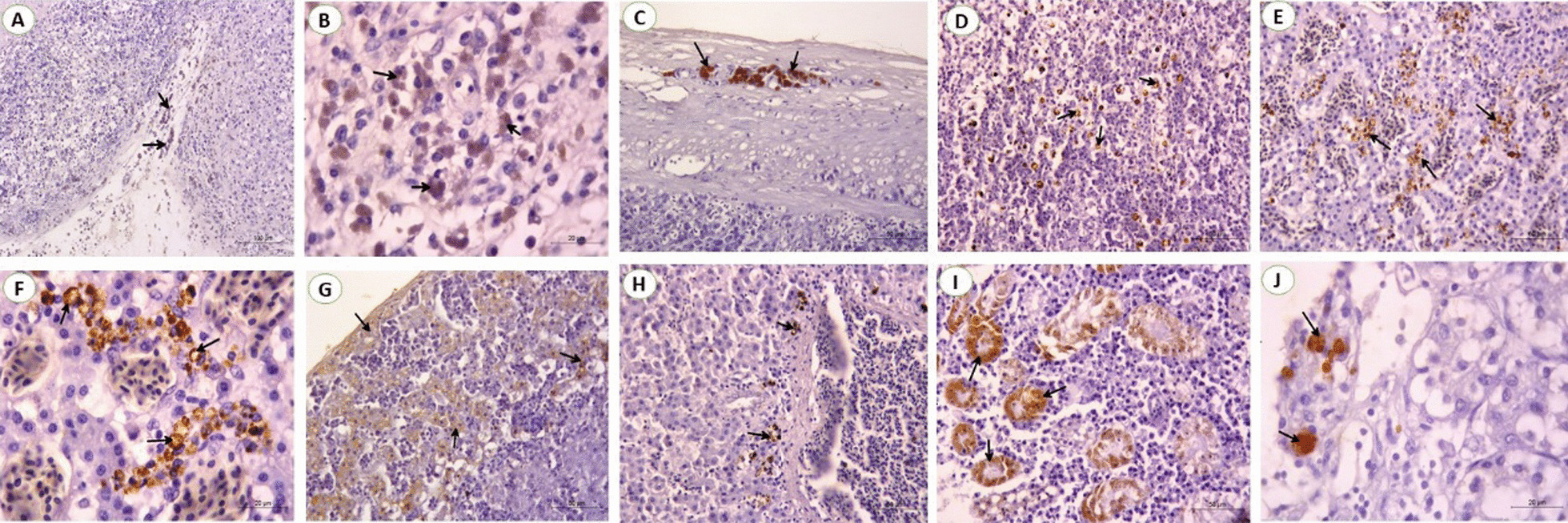
Immunoreactivity photomicrograph sections from the spleen, liver and kidney of broiler chickens naturally infected with myelocytomatosis; **A** Spleen showing staining of sub-capsular area that extending to the splenic trabeculae (arrow) and around lymphoid follicles (Scale bar: 100 µm), **B** Spleen showing staining of lymphoid follicle (Scale bar: 20 µm), **C** Spleen showing staining of connective tissue capsule (arrow) (Scale bar: 50 µm), and **D** Spleen showing staining of lymphoid tumor cells scattered within white pulp (Scale bar: 50 µm). **E** Liver showing staining of the hepatic parenchyma, especially around congested sinusoids (Scale bar: 50 µm), **F** Higher magnification of the previous photo showing antigen staining (arrow) (Scale bar: 20 µm), **G** Liver showing staining of the liver capsule and subcapsular area (Scale bar: 50 µm), **H** Liver showing staining around the central vein (Scale bar: 50 µm), **I** Kidney showing staining of the lining epithelium of renal tubules (arrow). (Scale bar: 50 µm), and **J** Kidney showing staining of inter-tubular area (Scale bar: 20 µm)

Immunohistochemistry was performed using the anti-ALV-J specific antibody to detect the myelocytomatosis positive signals (antigen) in different organs. The myelocytomatosis positive signals were indicated by the brown staining. The results showed that the ALV-J positive signals were mainly presented in the spleen, liver, and kidney. In the spleen, staining was greatest in the splenic trabeculae, subcapsular sinuses, and lymphoid follicles (Fig. 10A–D). In the liver, staining was intense in hepatocytes around congested blood vessels, infiltrated lymphoid cells, and subcapsular sinuses (Fig. 10E–H). In the kidney, a strong positive reaction in the lining epithelium of renal tubules, glomerular cells, and under the renal capsule was detected (Fig. 10I, J). In other tissues, there is no staining (Additional file 2).

## Discussion

ALV-J was a great threat causing huge economic losses in the poultry industry worldwide. Notably, ALV-J spread rapidly through poultry populations, and the emergence viral new strains has been spread rapidly among different countries thus, viral eradication is very necessary for commercial breeding flocks. ALV-J Infection is challenging to control without a vaccination program, thus the only obtainable control method is flock condemnation; certainly, China has managed and controlled ALV-J infection by cautiously selecting non-infected breeders for broiler and layer industry [25, 38, 39]. Interestingly, in Egypt, ALV-J spreads quickly during 2014 throughout Egyptian poultry flocks including native and foreign breeds of layers and ducks, with high mortality rates [16, 40]. Recently, sporadic cases of ALV-J were detected in Egypt in poultry and ducks with various genetic backgrounds [19, 41].

In this study, broiler flocks in highly poultry production three governorates (El-Sharqia, El-Dakahliya, and Al-Qalyubiyya) have shown general clinical symptoms with suspicion of viral natural infections. Also, the mortality rate was recorded at about 7% while the morbidity was about 20%; with non-specific necropsy except for off-white masses on the sternum and pelvis in some investigated birds. To the best of our knowledge, no research or report has been achieved mainly on ALV-J associated with myelocytomatosis in broiler flocks, in Egypt.

According to our findings, ALV-J CPE was observed after 72 h in inoculated CER cell culture as aggregation, rounding, ballooning, degeneration, and enormous detachments of cells. These results were in line with the CPE findings of [16, 28]. Also, [42] mentioned that cytopathic ALV strains inoculated in chicken embryo fibroblast cells have given CPE and cell detachment 3 days after infection. Concerning egg inoculation, ALV-J pathological lesions on SPF-ECE were mainly stunting, curling, anomalies, and enlarged liver. These lesions may be due to the ALV-J direct effect. Embryo mortality increased as the virus passing increased until the third passage. ALV-J has been reported to cause severe hemorrhage and embryo death within 4–5 days following embryo infection. These results came in accordance with [29] At the same time, results analysis obtained from the serological survey showed that 14 farms were positive for ALV-J (77.7%). The virus identification using antigen capture ELISA revealed that only 79 serum samples were positive for ALV-J (67.52%) based on the S/P ratio. These subsequent results are in agreement with [43, 44]. In addition, these findings are nearly in agreement with [16] who reported that the positive ELISA results of collected serum samples reach to 74.2% in broiler chickens. Moreover, [11] stated that ALV-J antibody titer was significantly (*P* < 0.01) increased in experimentally infected SPF chicks of one-day-old from the 3rd-month post-infection (mpi) till the 5th mpi (experiment end). Interestingly, our promising results confirm the high incidence of ALV-J in the currently examined farms indicating a vertical transmission of the causative virus. We first followed up on the ALV-J infected breeder hens and then confirmed by histopathology examination (unpublished data), after that all clinical specimens were collected from affected broiler flocks. However, [45] suggested that the hatching of one-day-old egg-type chicks with ALV-J-infected meat-type chicks in the same hatchery had contributed to horizontal infection.

Despite evidence of virus growth in SPF-ECE embryo and tissue culture, PCR is the most appropriate and rapid method to detect ALV-J; providing epidemiological data of various isolates prevalent in infected flocks periodically. Analysis of PCR assay with ALV-J specific primers from (the liver, spleen, and kidney) revealed that only 43 samples were positive with a percentage of 75%. Currently, the liver and spleen show very high tropism for ALV-J according to PCR results at 91.1% and 89.4%; respectively. These results are similar to those [46] who recorded the ALV-J high tropism in the different visceral organs, especially the liver and spleen. This result indicated that these samples were collected from viremic-tolerant chickens. Parallel to our results, [11, 16] reported that all examined samples obtained from the liver, spleen, and kidney indicated a positive reaction with ALV-J at 545 bp. Also, [19] mentioned that ALV-J infection was detected in the Lower Egypt layer farms in El-Qalyubia, El-Monofia, El-Gharbia, El-Behera, and El-Daqhlia governorates. Regarding our results, [41] reported also different rates of ALV-J infections in the different governorates (Gharbia, Damietta, Sharkia, and Dakahlia) based on qRT-PCR in collected breeder chickens and duck samples. Our results indicated that ALV-J is the main etiology of viral tumors in broilers at these three El-Sharqia, El-Dakahliya, and Al-Qalyubiya governorates. Regarding previous studies, the *gp85* encoding protein is highly evolved and capable of receptor-binding site, which plays a critical role in viral entry that determines the host ranges and tumor types [24, 25]. To investigate the genetic evolution of the *gp85* gene in our ALV-J isolates, they were sequenced, systematically analyzed then compared to other ALV-J reference sequences. The genetic characteristics of the reported Egyptian strains (ALV-J Dakahlia-2 and ALV-J Sharqia-1) were highly similar to other ALVs. Phylogenetic analysis indicated that the ALV-J Dakahlia-2 isolate has the highest genetically related to Egyptian isolates as ALV-EGY/YA 2021.3, ALV-EGY/YA 2021.4, ALV-EGY/YA 2021.14, and ALV-EGY/YA 2021.9 with nucleotide identity percentage 100%, 97%, 96%, 96%; respectively, and on the amino acid level were with 96%, 97%; 96%, 96%; respectively. Furthermore, ALV-J Sharqia-1 isolate is highly similar to other Egyptian strains like ALV-EGY/YA 2021.14, ALV-EGY/YA 2021.9, ALV-J isolate QL1, ALV-J isolate QL4, ALV-J isolate QL3, ALV-EGY/YA 2021.4 with nucleotide identity percentage 98%, 98%, 98%, 98%, 97%; respectively, and on the amino acid level were with 97%, 97%; 98%, 97%,97%, 95%; respectively. In particular, our previous results may suggest that these prevalent ALV-J isolates might be of the same sources or have similar ancestors. This reasonably elevated detection rate might be attributed to the vertical transmission of the. ALV-J infection. Meanwhile, ALV-J Dakahlia-2 and ALV-J Sharqia-1 isolates shared 73–75% homology with the American strain (ALJ-10022-2). Our findings are not in agreement with [47] who reported that the *gp85* of PK19SA01 shares 95.5% identity with the American strain. Also, ALV-J Dakahlia-2 and ALV-J Sharqia-1 isolates shared 91% -93% similarity with the American reference strain (ALJ-ADOL-7501). These subsequent results are not in agreement with [19] who mentioned that the Egyptian ALV-J isolates were similar to HPRS-1003 (prototype strain) with an identity percentage of 91.2–91.8%. Importantly, we can speculate that our Egyptian ALV-J strains might be introduced from America through chicken breeding. On the other hand, ALV-J isolates were distinctly apparent from Chinse isolates with 71% and 73% identity. These results are not parallel to [19] who stated that the nucleotide identity percentage of their Egyptian isolates was within the range of 88–94% when compared to Chinese reference strains.

The core region of the *gp85* gene contains five variable regions (hr1, hr2, vr1, vr2, and vr3) [48]. These regions (hr1, hr2, and vr3) are ALV-J receptor interaction determinants [49]. Previous studies revealed that the *gp85* gene tends to mutate as a result of immune pressure, causing a lot of changes in antigenic properties and virulence [20]. In the present study, no evidence of variations or amino acid mutations of our isolates in the *gp85* gene were detected in the putative variable regions, vr2. These current findings are similar to those [19] who recorded that no changes existed in the vr2 domain of the *gp85* gene of their isolates at the layer farms. In contrast, these results are not in agreement with [41] who reported 25 true SNPs among the five strains of chicken and duck breeders, in which only 5 SNPs lead to amino acid mutation. These amino acid substitutions might cause variations in the pathogenicity, oncogenicity, and ALV-J host range. Finally, our findings act as a warning that the ALV-J eradication is not disposable, so continuous monitoring is essential.

The histopathological picture besides the molecular characterization gave an accurate diagnosis for ALV-J. ALV-J could induce malignant or benign tumorigenic diseases and immunosuppressive responses in poultry such as hemangiomas, myelomas, and myelocytomatosis. Tumor development is a multi-step process representing the abnormal expression of an apoptotic gene, inactivation of tumor suppressor genes, or activation of proto-oncogenes [50]. Myelocytomatosis causes high economic losses in white meat-type breeder farms [51]. Furthermore, myelocytomatosis is a tumor disease in which tumor progress is a process that is complicated and related to many factors as genetic background, immune competence, and viral infection factors. The process of promoting carcinogenesis is still unknown, but the integration of the myelocytomatosis provirus may interfere with the function of the host endogenous gene [52, 53].

The immune suppression due to myelocytomatosis may involve atrophy of lymphoid organs, decreased mitogen-induced blastogenesis, and decreased antibody response [54]. Interestingly, the immune system alteration occurs as a result of cessation of B cell maturation in addition to a blockage in the development of T-suppressor cells, probably due to hindrance with functional IL-2 synthesis [55, 56] showed that ALV-J could induce lymphocyte apoptosis in immune organs, particularly in young chickens. Lymphocyte death increases susceptibility to other diseases.

Myelocytomas have a characteristic gross appearance in which myelocytes proliferate and soon overgrow the bone marrow. Tumors are formed by the expansion of marrow growth and may crowd through the bone and periosteum. They occur frequently on the surface of bone such as the costochondral junctions of the ribs and on the sternum and pelvis, these aforementioned lesions agree with [46, 57].

Histopathological evaluation of the liver concluded the presence of myeloid and lymphoid. The features of the liver lesions were aggregations of mature granulated myeloid cells. The neoplastic cells replaced the hepatocytes with relative atrophy of the surrounding cells. The same findings were recorded by [11, 37]. Meanwhile, focal aggregations of lymphoblastic cells were detected in the hepatocyte. Furthermore, In the liver, the brown granules resembled a positive reaction for specific viral particles present in the Kupffer cells and lymphocytes as well as erythroblasts; this supports the previous findings which were also confirmed by detecting the virus from liver tissues using PCR. These results agree with [5, 41, 53, 58, 59].

In contrast, the microscopic picture of spleen sections showed that ererea a pleomorphic lymphoid population surrounding arteriole. By Giemsa stain, myeloid cells appear obviously with their eosinophilic granules. Viral antigen was greatest in the splenic trabeculae, subcapsular sinuses, and lymphoid follicles by IHC. These results were supported by [37] who recorded ALV-J positive signals in the erythroblast cytoplasm, spleen, lung, and other tissues especially rich in blood.

The microscopic examination of the kidney revealed multifocal neoplastic aggregations of granular myeloid cells. The tumor cells were aggregated between the degenerated renal tubules and around the congested blood vessels. Granulated myeloid cells were seen infiltrating the interstitial tissue causing pressure atrophy and loss of some renal tubules when stained with Giemsa stain, that result was confirmed by [41, 58, 60, 61].

The proliferation of granulated myelocytes was detected in the bone marrow and the periosteum of the sternum. Proliferation begins in the bone marrow of epiphysis. The Myelocytes invaded from the bone marrow to periosteal areas through Haversian and Volkmann’s canals. Myelocyte proliferation was also detected in the bone marrow. Additionally, there is a wall thickening and lumen narrowing of the sternal bone together with dentation in the periosteum which is considered as osteopetrosis. Finally, ALV-J tropism for chicken bone marrow cells, and induces their neoplastic transformation [62]. This finding agrees with that recorded by [11, 63, 64].

## Conclusions

We first isolated two ALV-J strains associated with myelocytomatosis from broiler flocks, in Egypt. In summary, the circulating ALV-J infection associated with myelocytomatosis during 2023 in broiler flocks at different localities of Egypt was diagnosed through PCR technique, serological assay, molecular sequencing approaches, pathological, and immunohistochemical examinations. ALV-J causes neoplastic diseases in broiler flocks, with the highest rate of infection presented in these governorates as El-Sharqia, El-Dakahliya, and Al-Qalyubiyya. In addition, the ALV-J *gp85* gene evolution of our isolate (Dakahlia-2, identified as subgroup II) have the highest genetically related to ALV-EGY/YA 2021.3, ALV-EGY/YA 2021.4, and ALV-EGY/YA 2021.14 with nucleotide identity percentage 100%, 97%, 96%; respectively, and on the amino acid level were with 96%, 97%; 96%; respectively. Moreover, ALV-J Sharqia-1 isolate is highly similar to ALV-EGY/YA 2021.14, ALV-EGY/YA 2021.9, and ALV-J isolate QL1 with nucleotide identity percentage of 98%, and on the amino acid level were with 97%, 97%; 98%; respectively. Our Egyptian ALV-J isolates (ALV-J Dakahlia-2 and ALV-J Sharqia-1) were submitted to Genbank with accession numbers (OR509852–OR509853). The phylogenetic analysis based on the nucleotide and deduced amino acid sequences of the *gp85* gene showed no evidence of variations or amino acid mutations in the putative variable domains, vr2. Currently, no vaccinations or treatments for ALV are presented and such ALV still threatens the local poultry industry. This reminds us to eradicate the positive cases, strengthen the breeder introduction detection, and apply periodic molecular monitoring for all recent Egyptian strains. Furthermore, the whole genome sequencing of these isolates is recommended to detect both the pathogenicity and antigenicity of these circulating ALV-J strains.

### Supplementary Information

**Additional file 1**. Original data of S/P ratio of sera collected from diseased and healthy broiler flocks for ELISA test of ALV-J.

**Additional file 2**. Original photos before cropping of PCR assay, ECE inoculation, Macroscopical findings of ALV-J, pathological findings of liver, kidney, spleen, Bone and myelocytoma, and Immunohistochemical findings of ALV-J.

## Data Availability

Not applicable.
