# Supplementary material for: Immunopathological investigation and genetic evolution of Avian leukosis virus Subgroup-J associated with myelocytomatosis in broiler flocks in Egypt
Source: Virol J. 2024 Apr 10;21:83. doi: 10.1186/s12985-024-02329-7 (PMC11005230; doi:10.1186/s12985-024-02329-7)
Supplement: Supplementary file 1 — Additional file 1. Original data of S/P ratio of sera collected from diseased and healthy broiler flocks for ELISA test of ALV-J. [file 12985_2024_2329_MOESM1_ESM.docx]

| Sample number | *S/P ratio of sera collected from diseased  broiler flocks | S/P ratio of healthy broiler broiler flocks |
| --- | --- | --- |
| 1 | 0.8 | 0.4 |
| 2 | 0.7 | 0.5 |
| 3 | 1.1 | 0.3 |
| 4 | 0.8 | 0.5 |
| 5 | 0.6 | 0.3 |
| 6 | 0.9 | 0.3 |
| 7 | 0.8 | 0.5 |
| 8 | 1.1 | 0.4 |
| 9 | 0.9 | 0.3 |
| 10 | 0.6 | 0.2 |
| 11 | 0.8 | 0.4 |
| 12 | 0.9 | 0.3 |
| 13 | 0.9 | 0.5 |
| 14 | 0.7 | 0.3 |
| 15 | 0.6 | 0.4 |
| 16 | 1.1 | 0.4 |
| 17 | 1.2 | 0.5 |
| 18 | 0.8 | 0.4 |
| 19 | 0.9 | 0.3 |
| 20 | 1 | 0.5 |
| 21 | 0.6 | 0.4 |
| 22 | 0.7 | 0.3 |
| 23 | 1.2 | 0.5 |
| 24 | 1.1 | 0.4 |
| 25 | 0.9 | 0.3 |
| 26 | 0.8 | 0.3 |
| 27 | 1.3 | 0.4 |
| 28 | 1 | 0.5 |
| 29 | 0.8 | 0.4 |
| 30 | 1.1 | 0.5 |
| 31 | 1.2 | 0.3 |
| 32 | 0.9 | 0.5 |
| 33 | 0.8 | 0.4 |
| 34 | 0.6 | 0.5 |
| 35 | 0.7 | 0.4 |
| 36 | 1.1 | 0.5 |
| 37 | 1.2 | 0.3 |
| 38 | 0.9 | 0.4 |
| 39 | 1.1 | 0.3 |
| 40 | 0.6 | 0.4 |
| 41 | 1.2 | 0.5 |
| 42 | 0.8 | 0.4 |
| 43 | 0.9 | 0.3 |
| 44 | 1.2 | 0.2 |
| 45 | 0.8 | 0.3 |
| 46 | 1.3 | 0.4 |
| 47 | 1.1 | 0.5 |
| 48 | 0.6 | 0.3 |
| 49 | 1.2 | 0.5 |
| 50 | 1.3 | 0.3 |
| 51 | 1.4 | 0.5 |
| 52 | 0.8 | 0.4 |
| 53 | 0.6 | 0.4 |
| 54 | 0.8 | 0.4 |
| 55 | 1.1 | 0.5 |
| 56 | 1.2 | 0.3 |
| 57 | 0.8 | 0.2 |
| 58 | 0.8 | 0.3 |
| 59 | 0.6 | 0.5 |
| 60 | 0.6 | 0.4 |
| 61 | 0.9 | 0.5 |
| 62 | 1.1 | 0.3 |
| 63 | 0.8 | 0.5 |
| 64 | 1.3 | 0.3 |
| 65 | 1.2 | 0.4 |
| 66 | 0.9 | 0.5 |
| 67 | 0.7 | 0.4 |
| 68 | 0.9 | 0.3 |
| 69 | 1.1 | 0.5 |
| 70 | 1.3 | 0.5 |
| 71 | 0.6 | 0.3 |
| 72 | 0.9 | 0.5 |
| 73 | 1.1 | 0.3 |
| 74 | 1.3 | 0.4 |
| 75 | 0.8 | 0.2 |
| 76 | 0.9 | 0.3 |
| 77 | 1.3 | 0.4 |
| 78 | 1 | 0.3 |
| 79 | 0.8 | 0.4 |
| 80 | 0.7 | 0.5 |
| 81 | 0.6 | 0.5 |
| 82 | 1 | 0.3 |
| 83 | 1.2 | 0.2 |
| 84 | 0.8 | 0.4 |
| 85 | 1.3 | 0.5 |
| 86 | 1.1 | 0.3 |
| 87 | 0.8 | 0.5 |
| 89 | 0.6 | 0.4 |
| 90 | 0.9 | 0.3 |
| 91 | 1.1 | 0.2 |
| 92 | 1.2 | 0.5 |
| 93 | 0.8 | 0.4 |
| 94 | 1.4 | 0.3 |
| 95 | 0.6 | 0.5 |
| 96 | 1.3 | 0.2 |
| 97 | 1.1 | 0.3 |
| 98 | 0.7 | 0.5 |
| 99 | 0.9 | 0.5 |
| 100 | 1.1 | 0.4 |
| 101 | 1.3 | 0.3 |
| 102 | 0.8 | 0.2 |
| 103 | 0.9 | 0.3 |
| 104 | 1.1 | 0.5 |
| 105 | 1.3 | 0.5 |
| 106 | 0.8 | 0.4 |
| 107 | 0.9 | 0.3 |
| 108 | 1.1 | 0.2 |
| 109 | 0.6 | 0.5 |
| 110 | 0.8 | 0.4 |
| 111 | 0.7 | 0.2 |
| 112 | 1.1 | 0.5 |
| 113 | 0.8 | 0.3 |
| 114 | 1.3 | 0.5 |
| 115 | 1.4 | 0.2 |
| 116 | 1.1 | 0.3 |
| 117 | 0.8 | 0.4 |
| **Mean** | **0.95** | **0.38** |
| **S.D** | **0.23** | **0.10** |
