# Supplementary figures and images for: Immunopathological investigation and genetic evolution of Avian leukosis virus Subgroup-J associated with myelocytomatosis in broiler flocks in Egypt
Source: Virol J. 2024 Apr 10;21:83. doi: 10.1186/s12985-024-02329-7 (PMC11005230; doi:10.1186/s12985-024-02329-7)

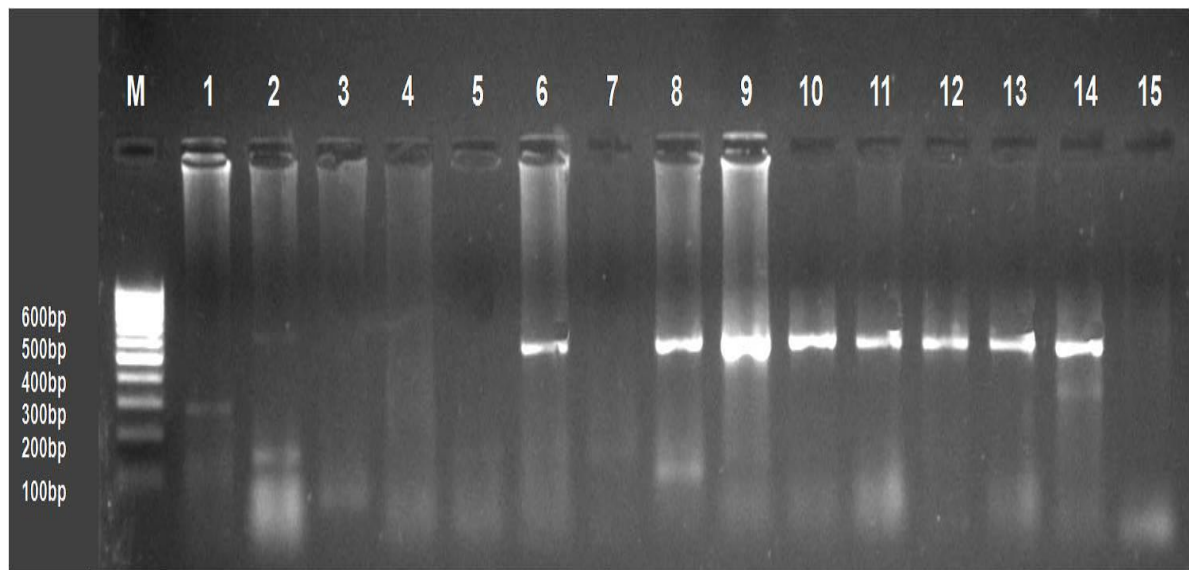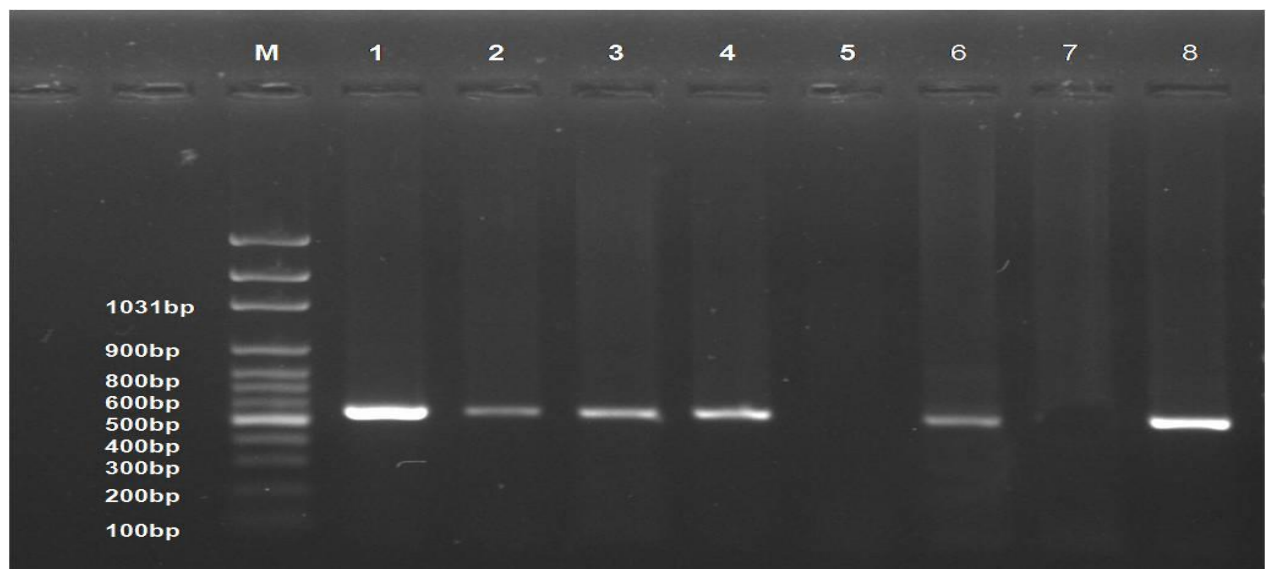

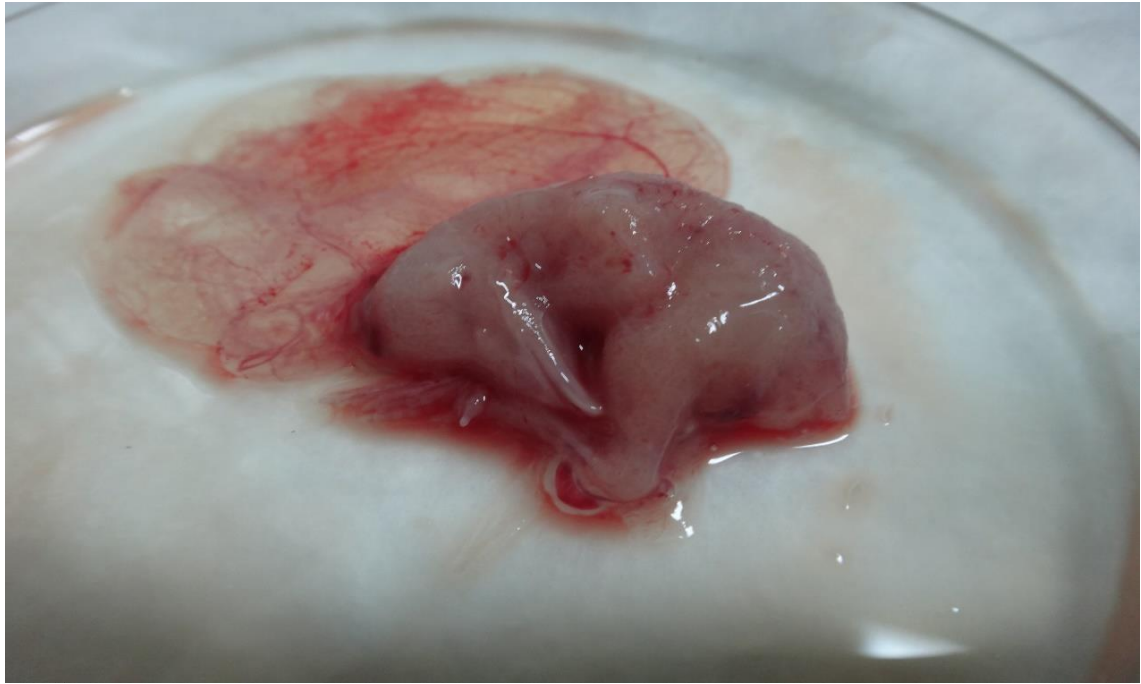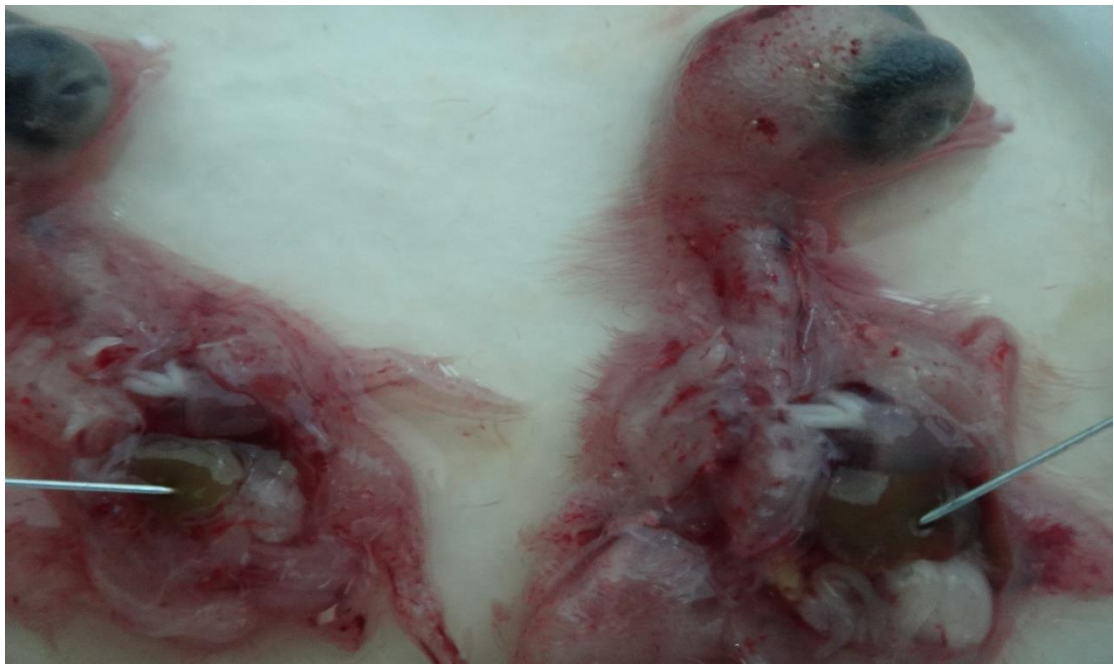

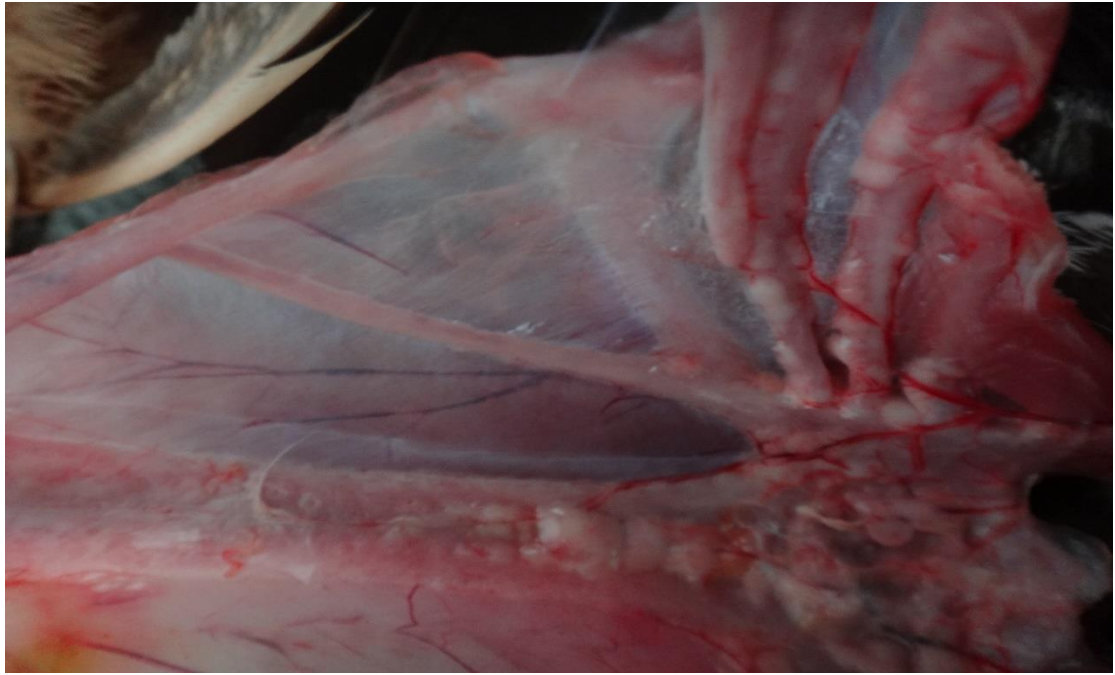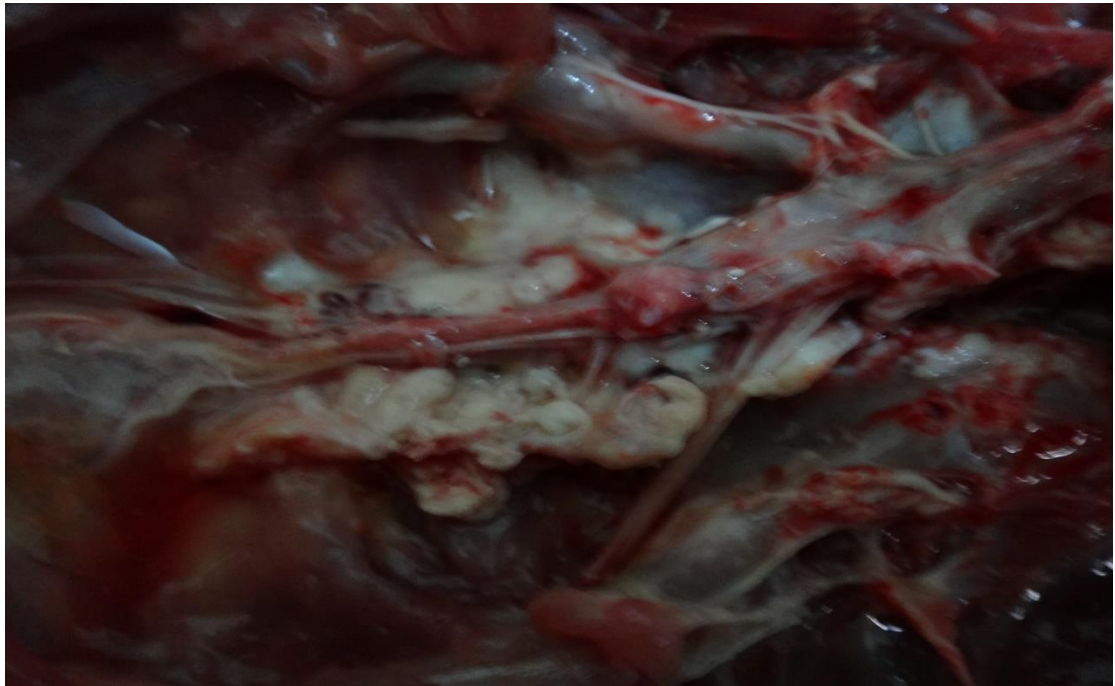

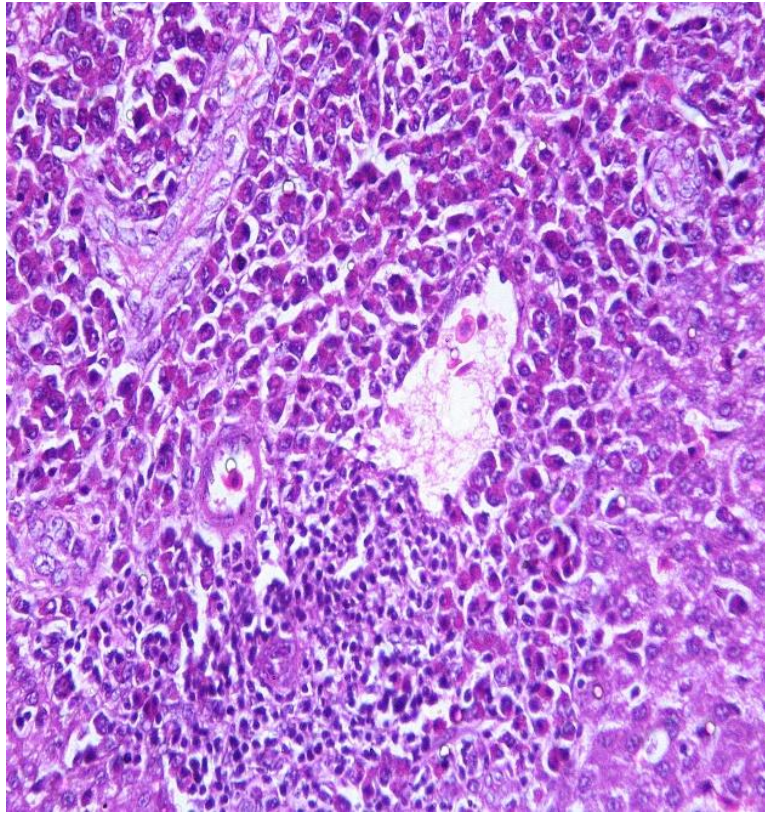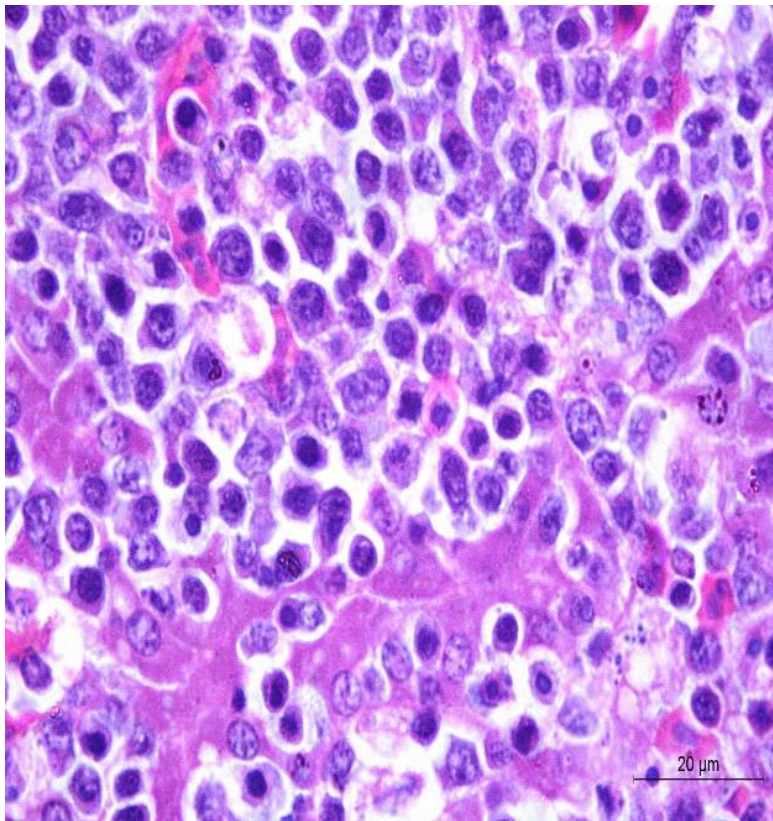

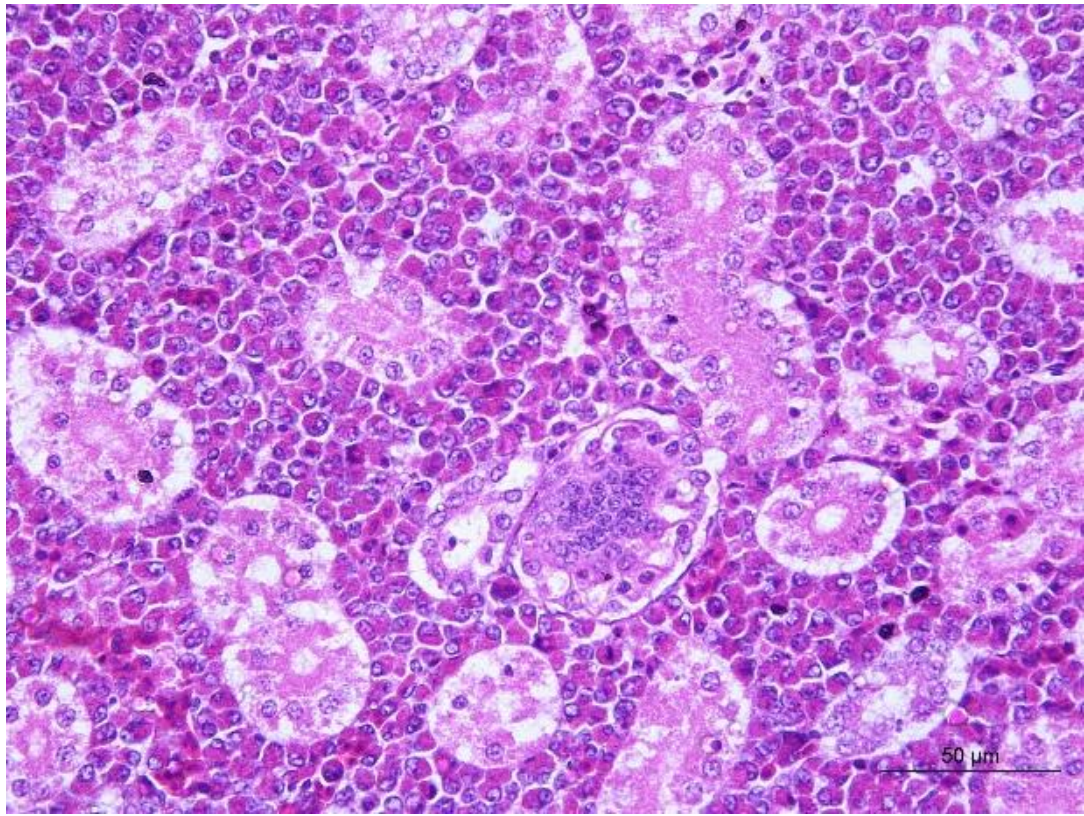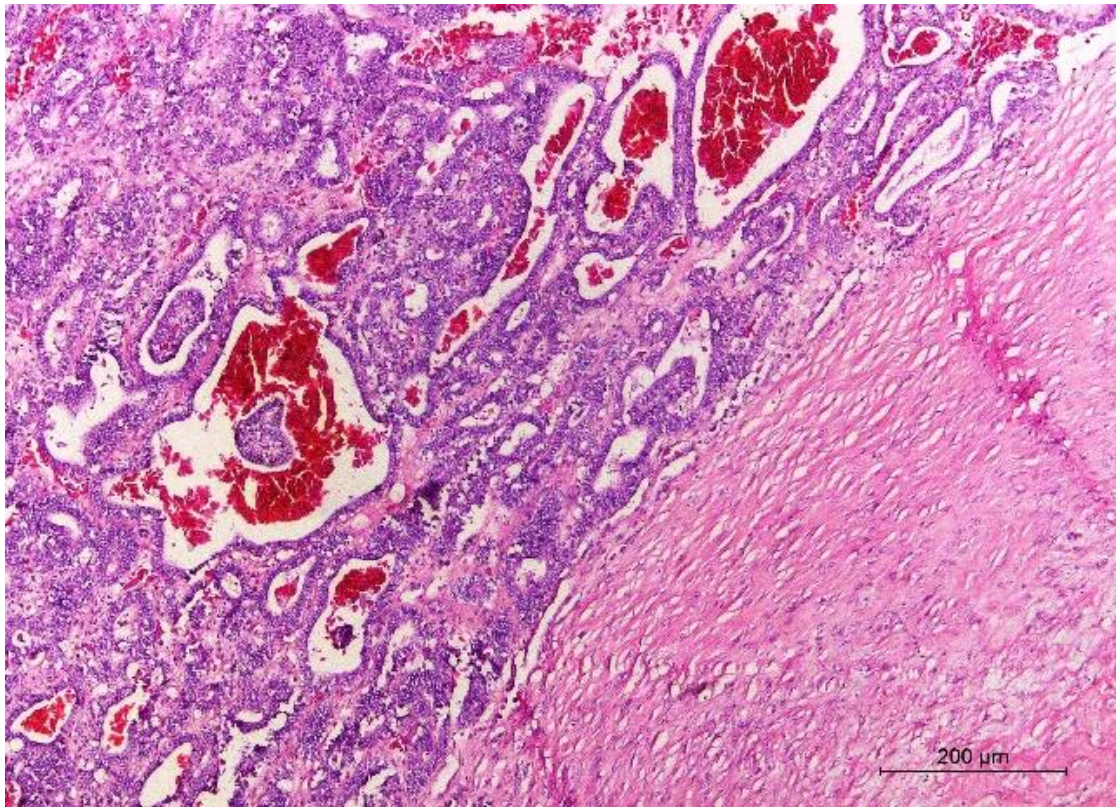

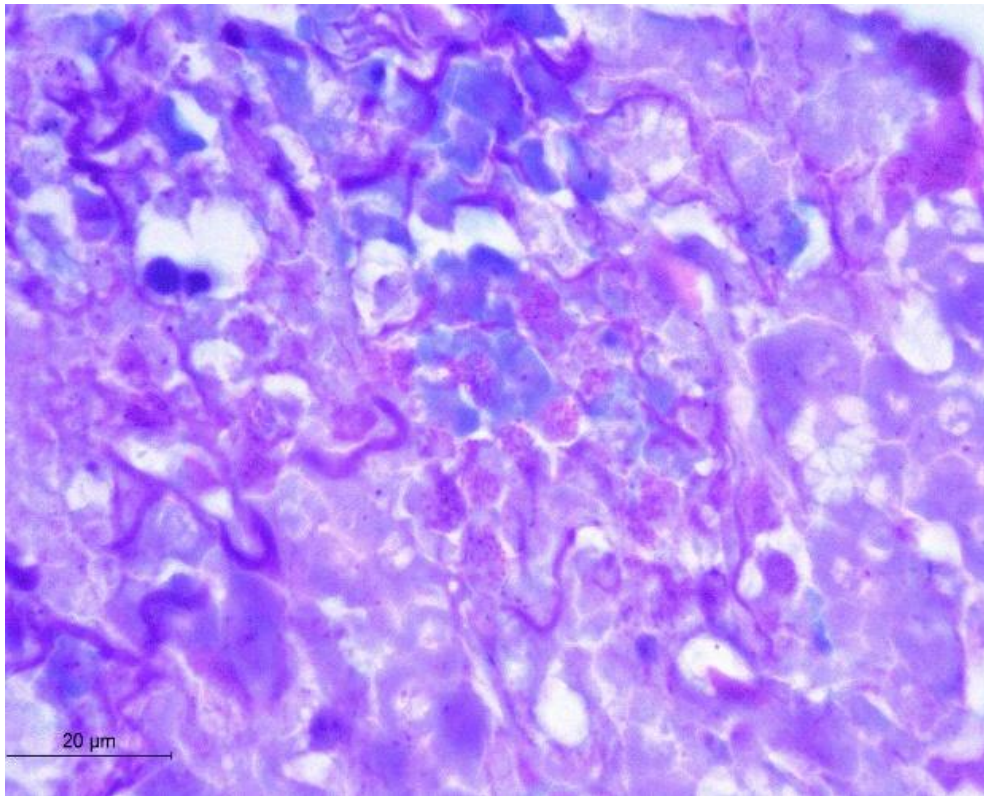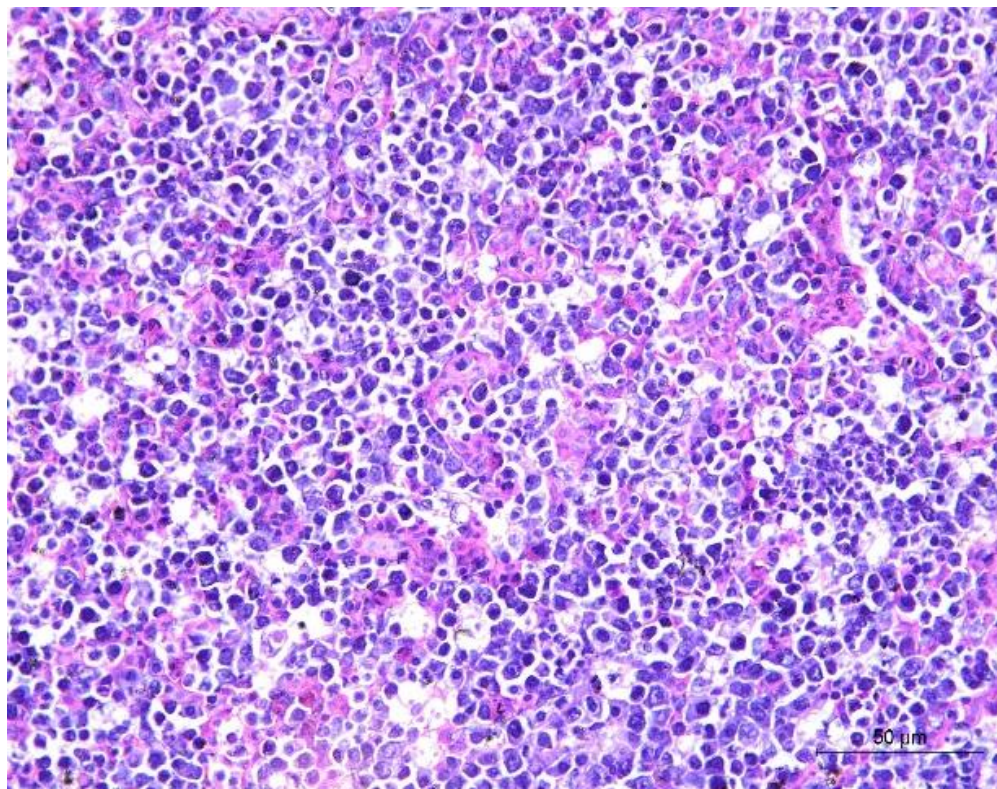

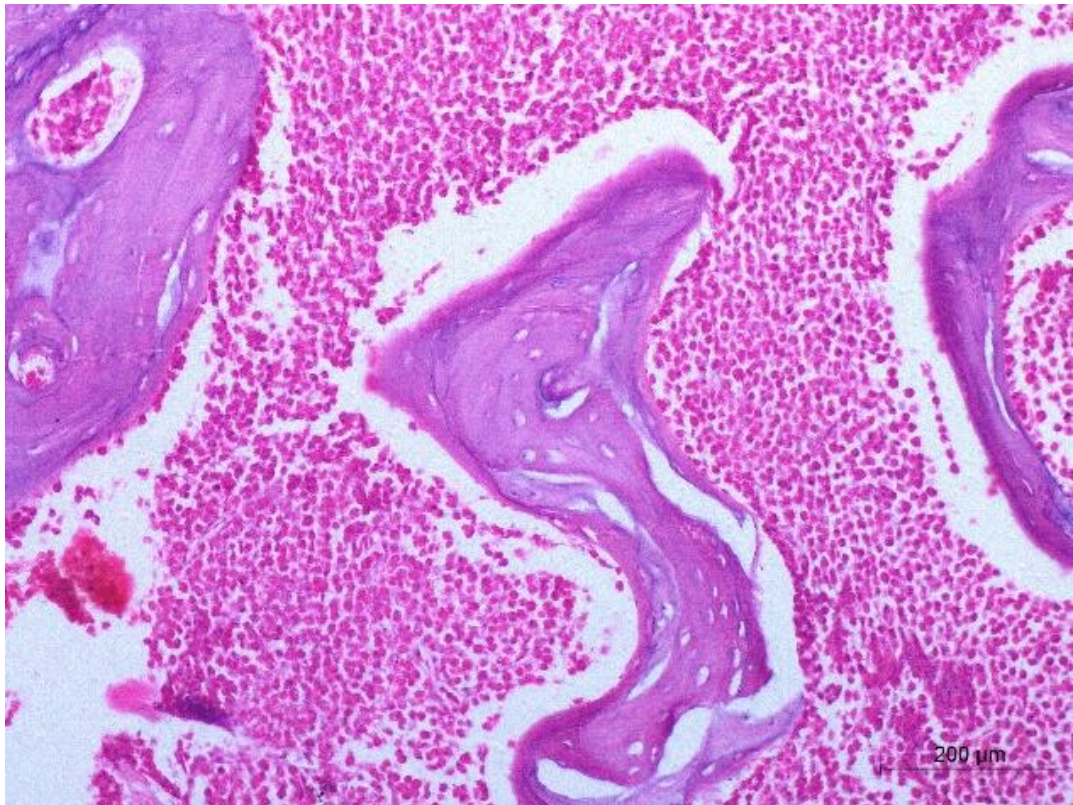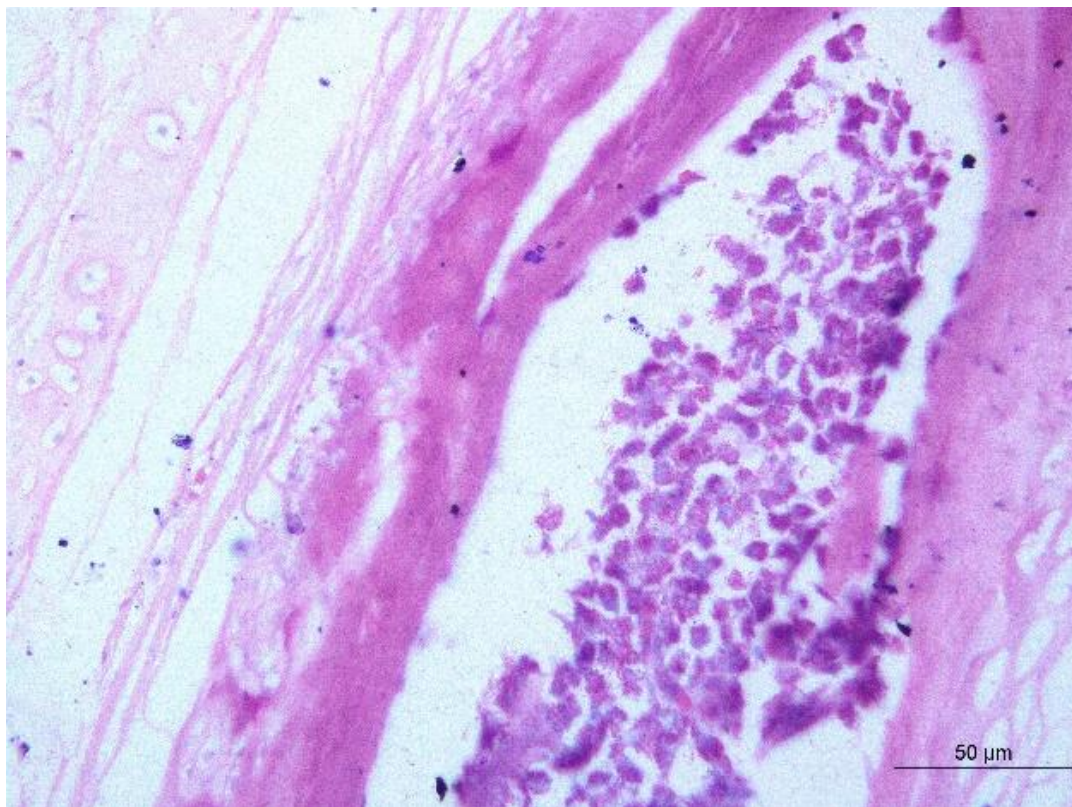

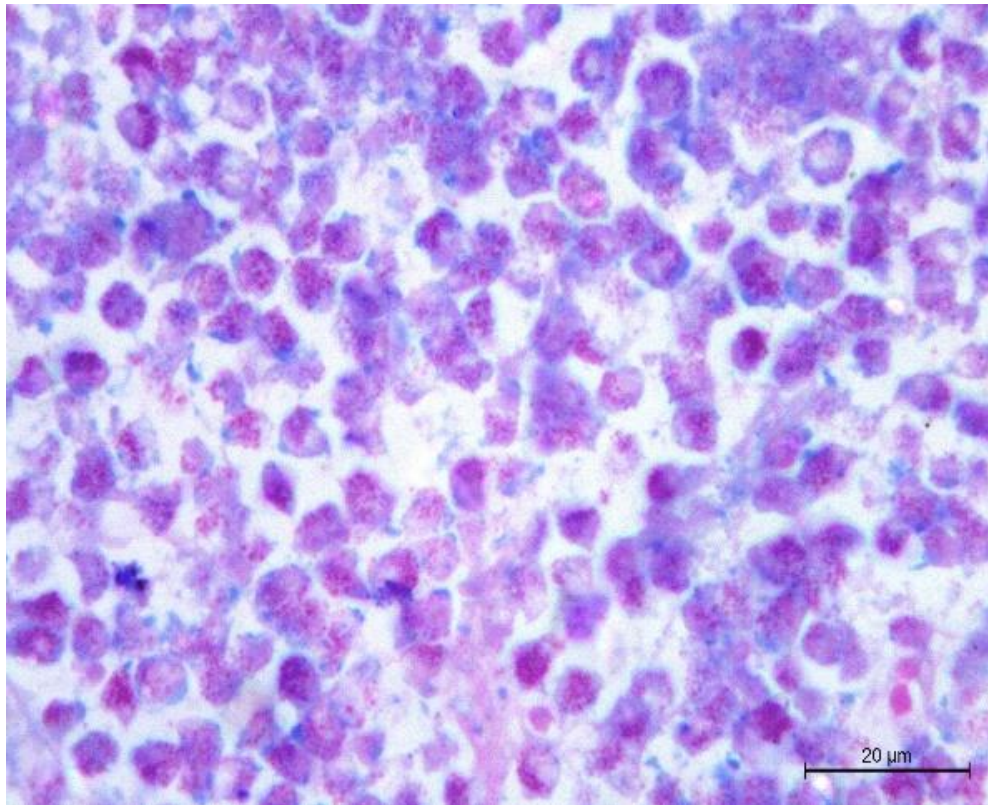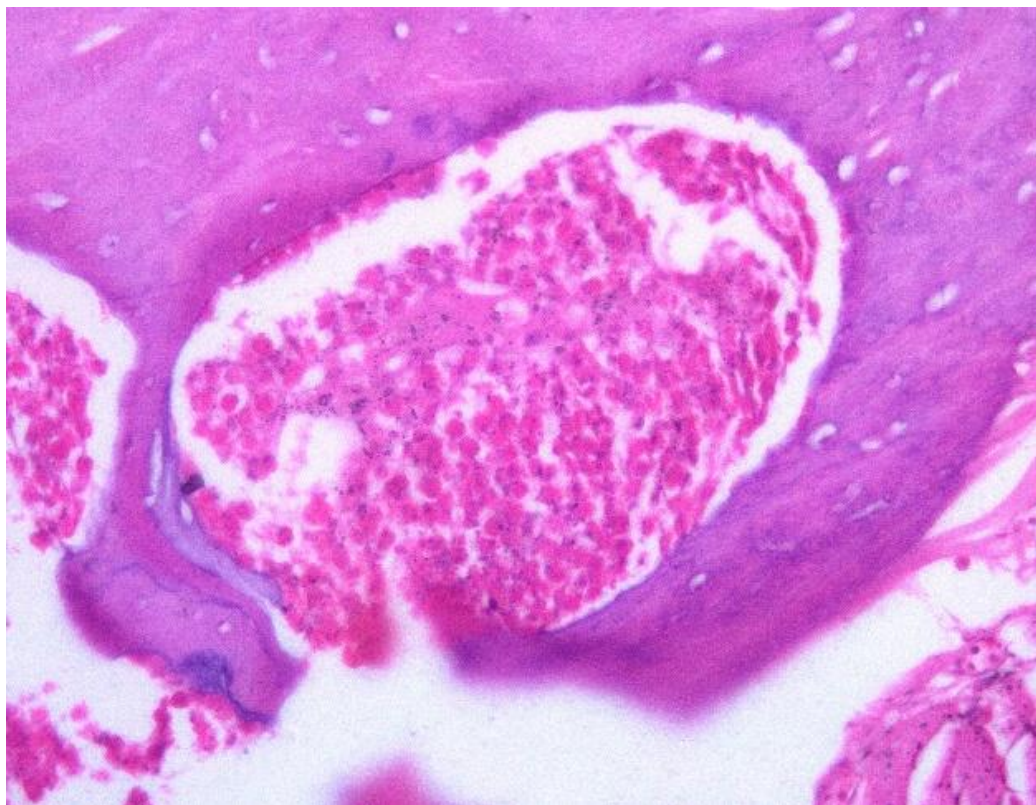

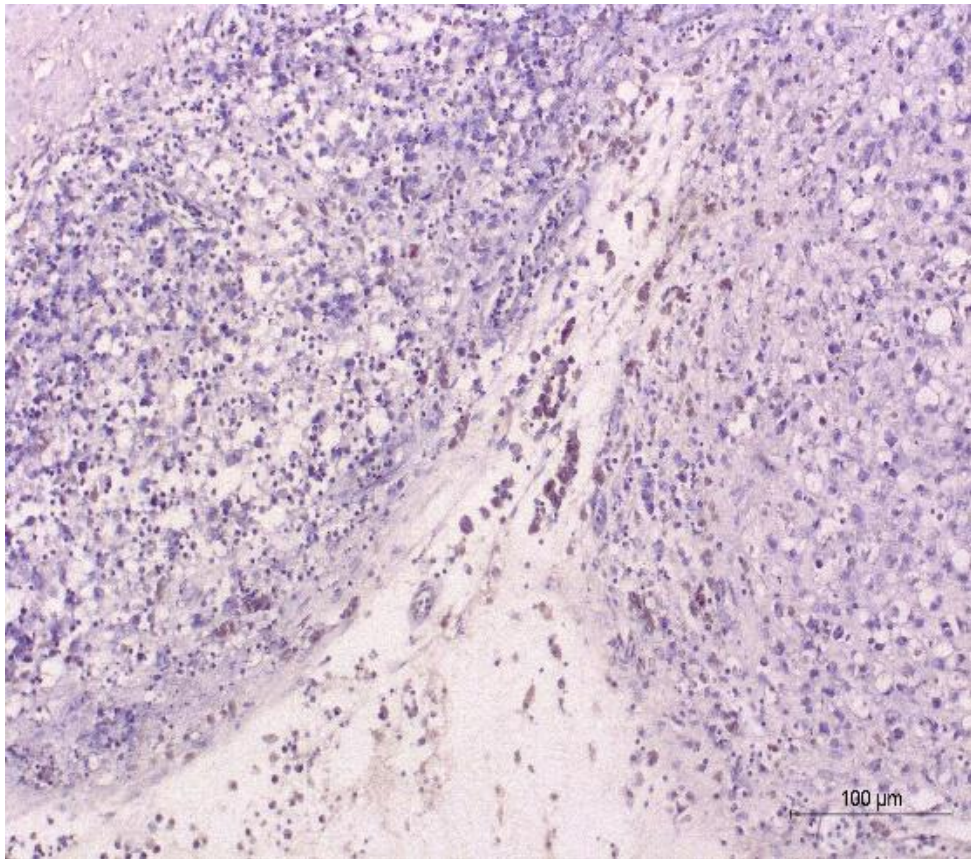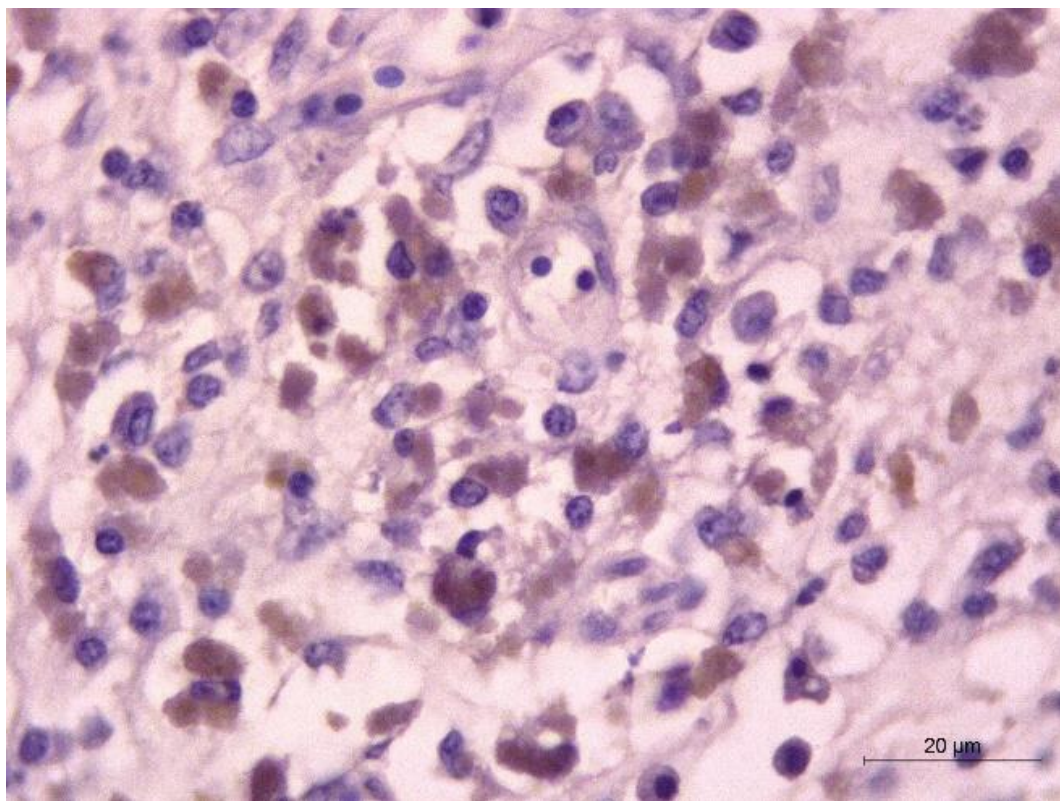

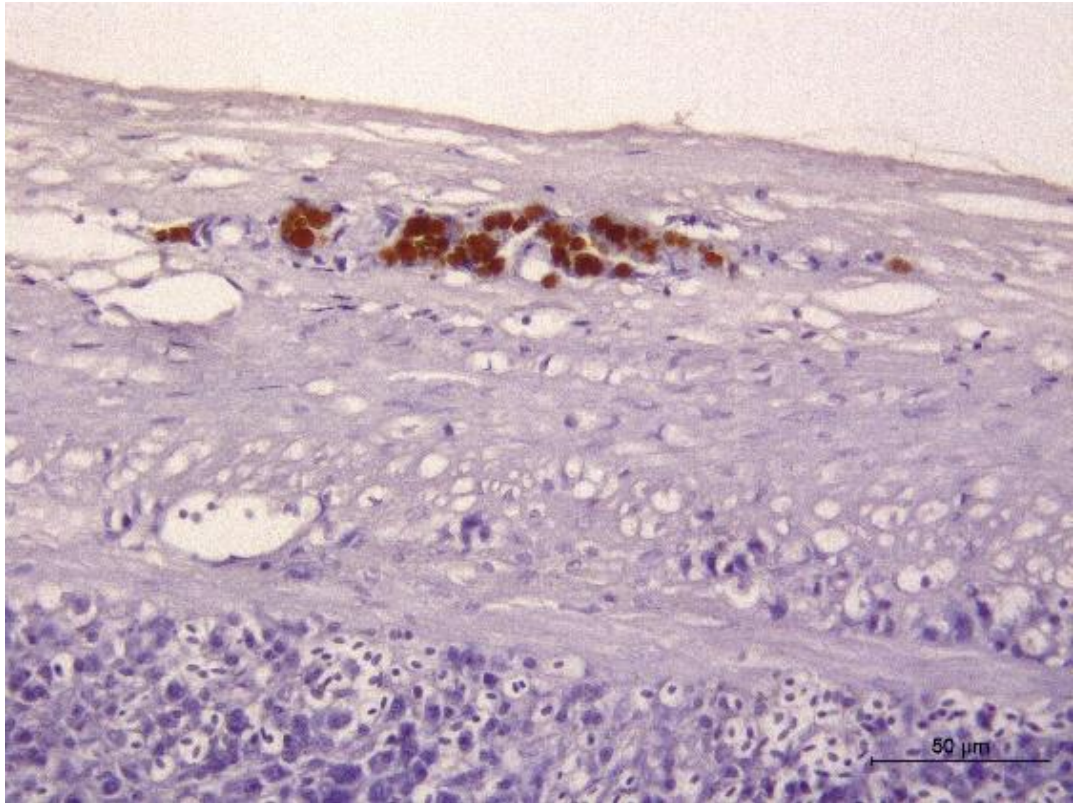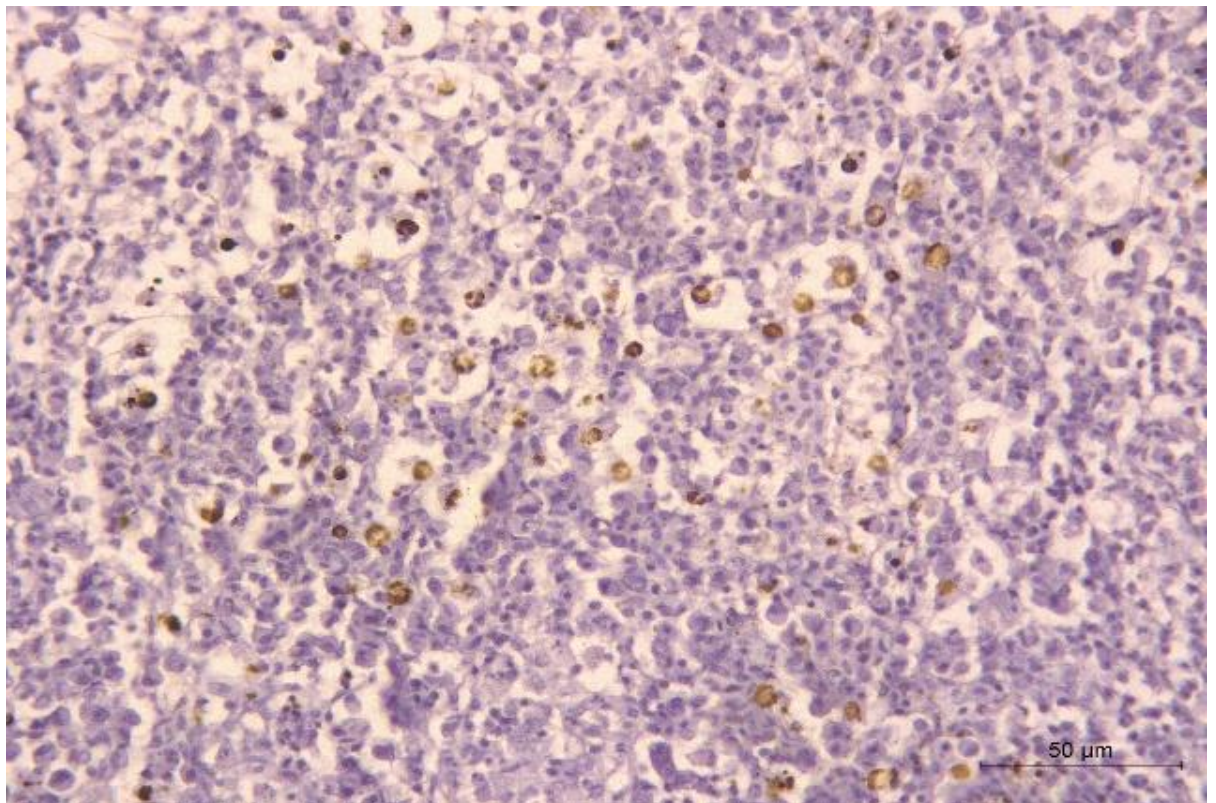

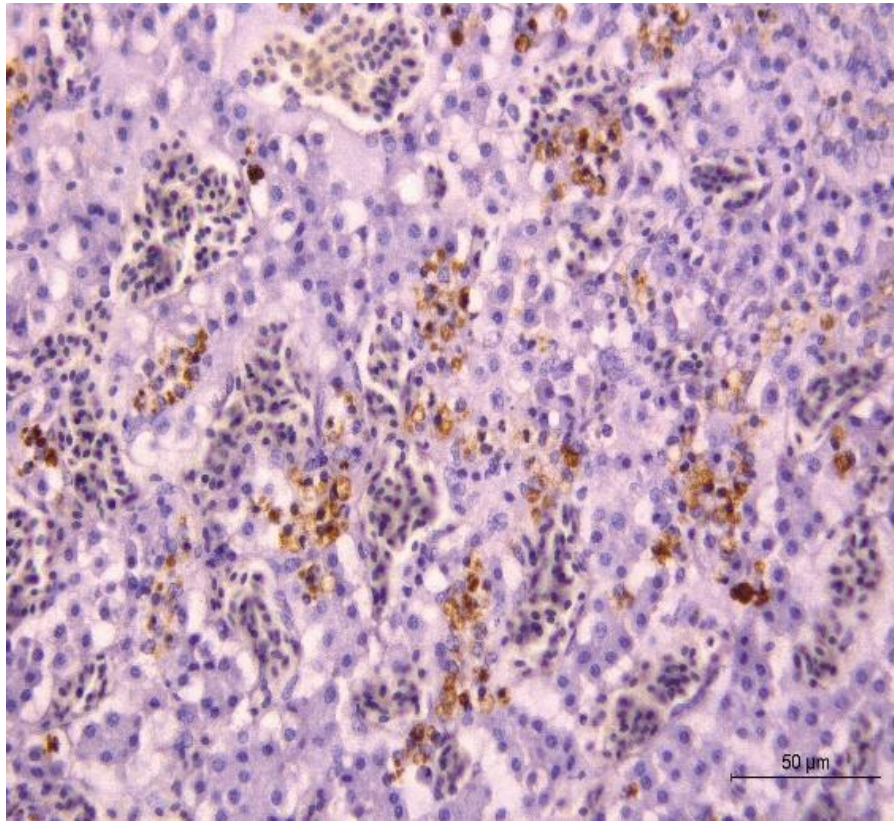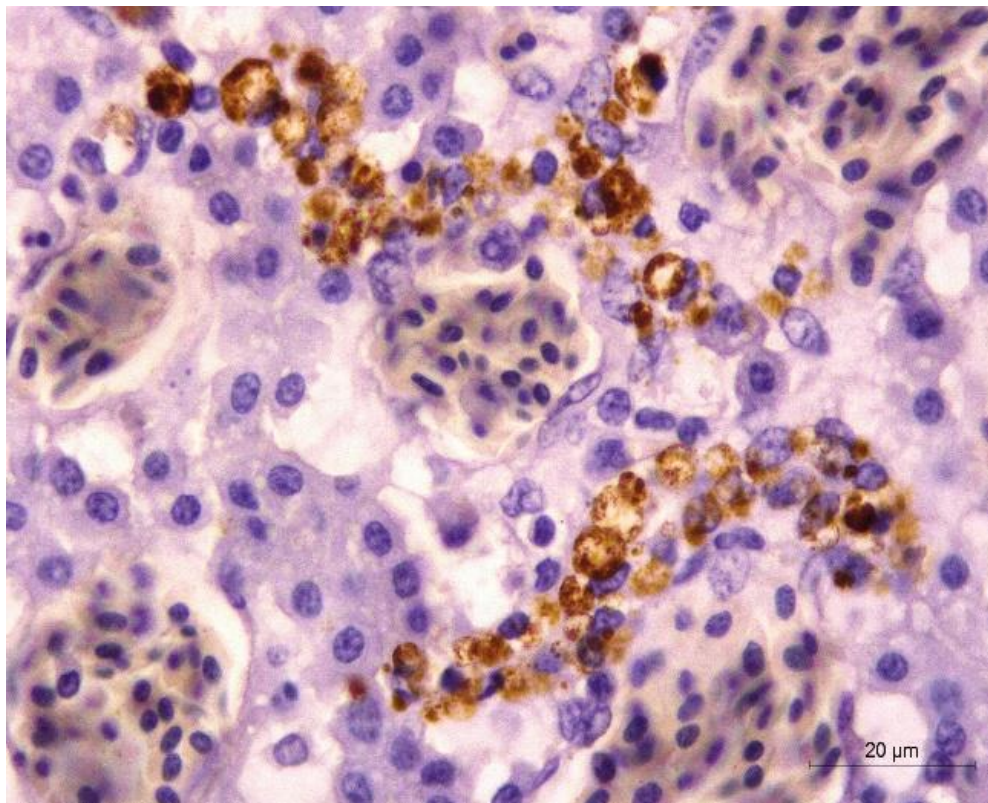

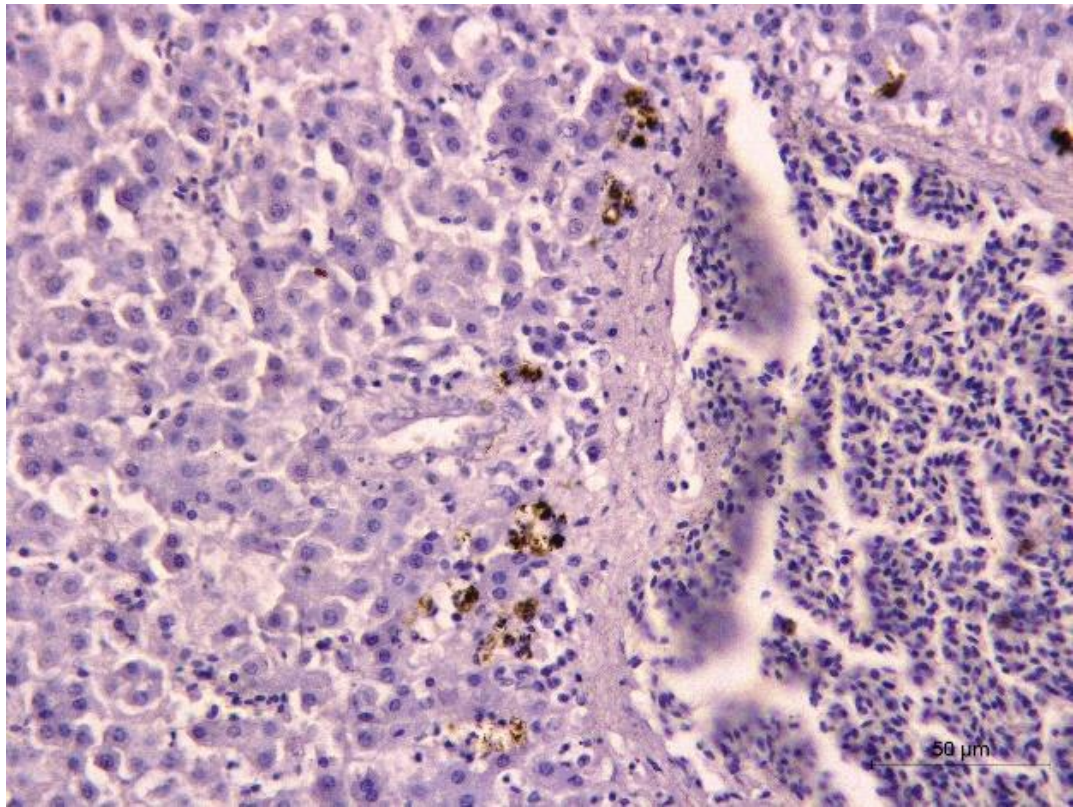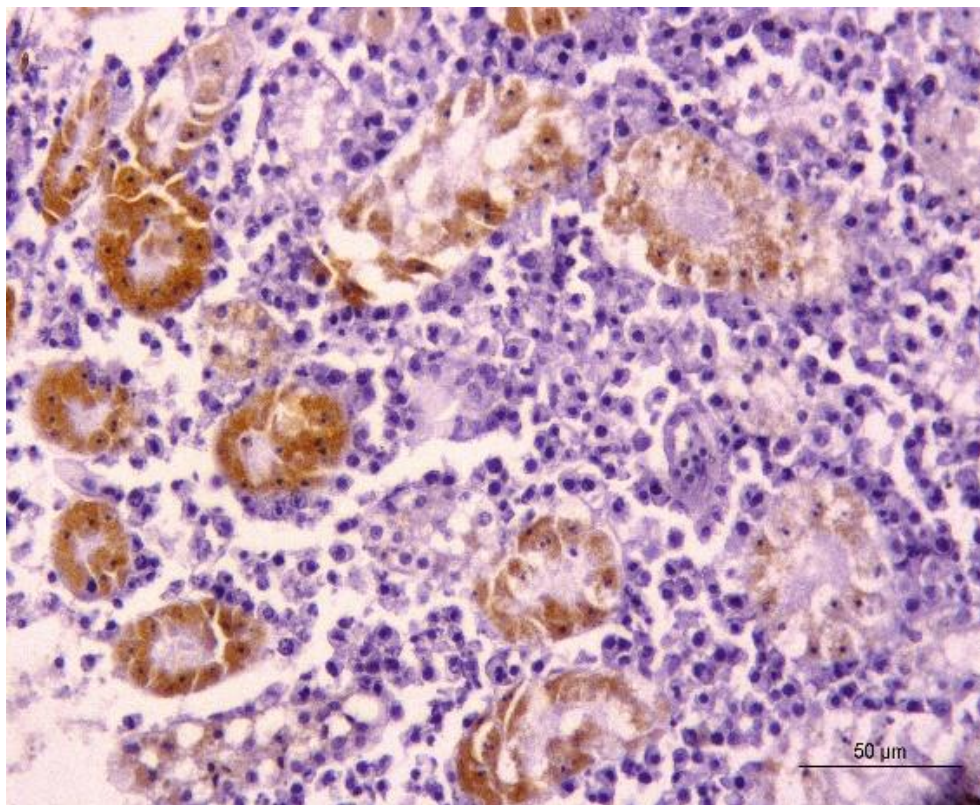

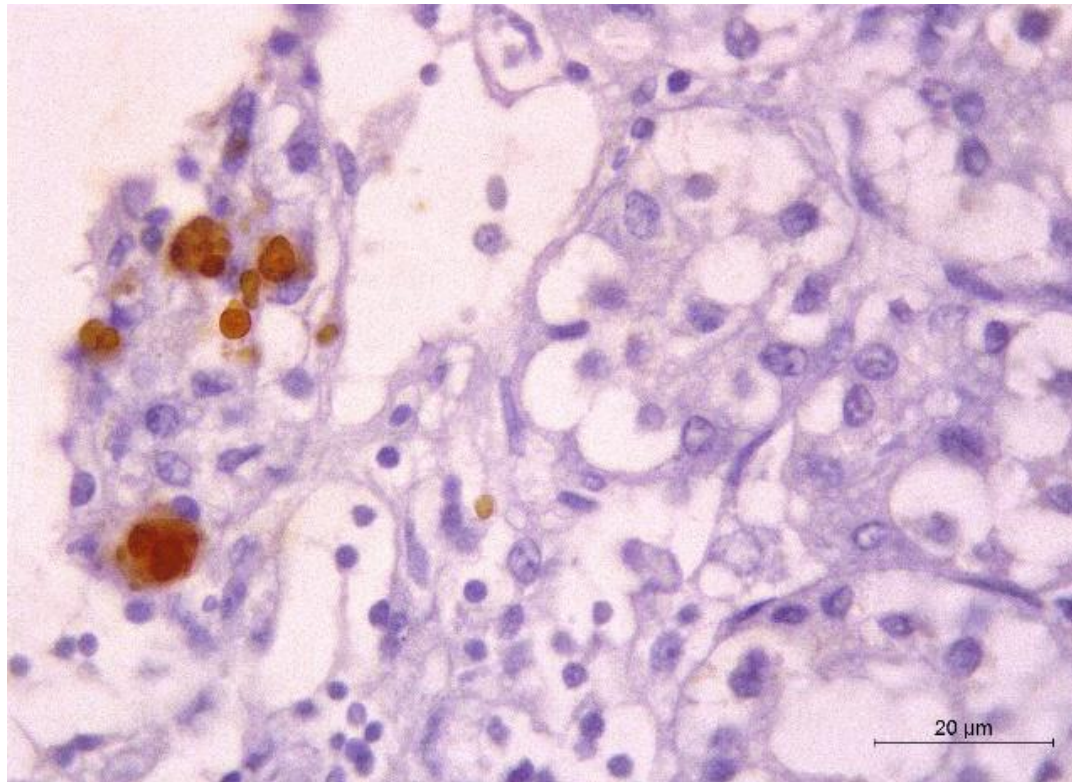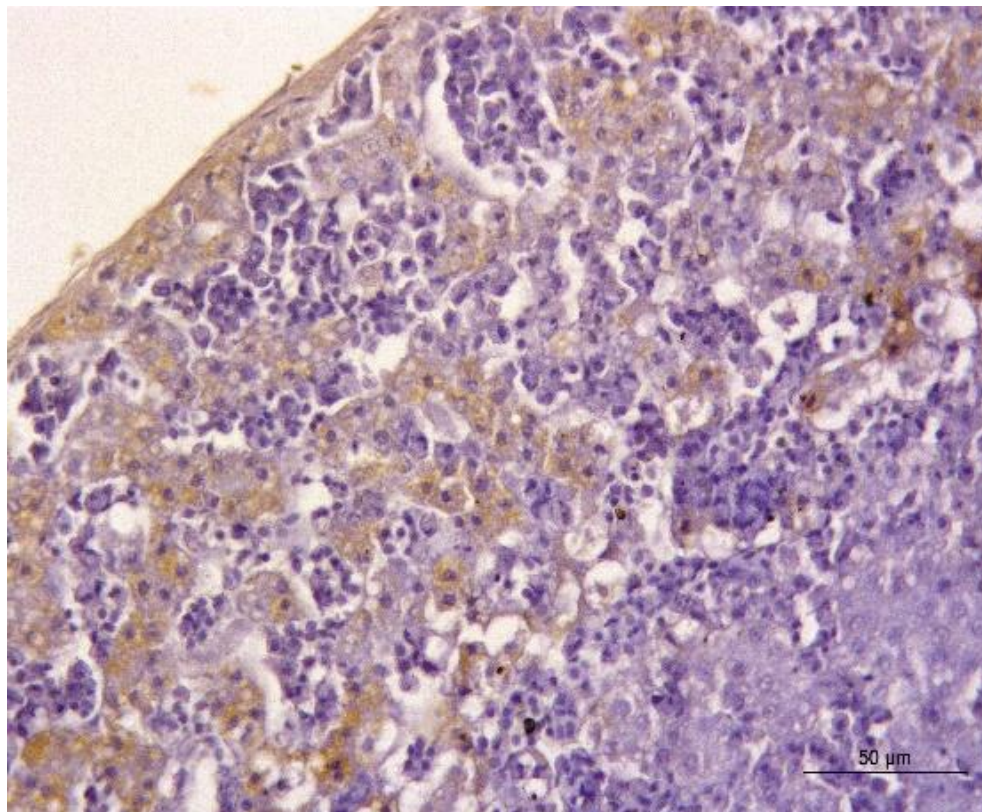

Supplement: Supplementary file 2 — Additional file 2. Original photos before cropping of PCR assay, ECE inoculation, Macroscopical findings of ALV-J, pathological findings of liver, kidney, spleen, Bone and myelocytoma, and Immunohistochemical findings of ALV-J. [file 12985_2024_2329_MOESM2_ESM.pdf]
